# A Comprehensive Review on the Valorization of Bioactives from Marine Animal By-Products for Health-Promoting, Biofunctional Cosmetics

**DOI:** 10.3390/md23080299

**Published:** 2025-07-26

**Authors:** Sofia Neonilli A. Papadopoulou, Theodora Adamantidi, Dimitrios Kranas, Paschalis Cholidis, Chryssa Anastasiadou, Alexandros Tsoupras

**Affiliations:** 1 Hephaestus Laboratory, School of Chemistry, Faculty of Sciences, Democritus University of Thrace, Kavala University Campus, St. Lucas, 65404 Kavala, Greece; sneopap@gmail.com (S.N.A.P.); theodoradamantidi@gmail.com (T.A.); dikrana@chem.duth.gr (D.K.); paoholi@chem.duth.gr (P.C.); 2 Fisheries Research Institute, Hellenic Agricultural Organization-DIMITRA (ELGO-DIMITRA), Nea Peramos, 64007 Kavala, Greece; anastasiadou@elgo.gr

**Keywords:** marine by-products, mollusk by-products, crustacean waste, fish by-products, extraction methods, bioactives, polar lipids, marine cosmetics

## Abstract

In recent decades, there has been a marked surge in the development of marine-by-product-derived ingredients for cosmetic applications, driven by the increasing demand for natural, sustainable, and high-performance formulations. Marine animal by-products, particularly those from fish, crustaceans, and mollusks, represent an abundant yet underutilized source of bioactive compounds with notable potential in cosmeceutical innovation. Generated as waste from the fishery and seafood-processing industries, these materials are rich in valuable bioactives, such as chitosan, collagen, peptides, amino acids, fatty acids, polar lipids, lipid-soluble vitamins, carotenoids, pigments, phenolics, and mineral-based substrates like hydroxyapatite. Marine by-product bioactives can be isolated via several extraction methods, and most importantly, green ones. These compounds exhibit a broad spectrum of skin-health-promoting effects, including antioxidant, anti-aging, anti-inflammatory, antitumor, anti-wrinkle, anti-hyperpigmentation, and wound-healing properties. Moreover, applications extend beyond skincare to include hair, nail, and oral care. The present review provides a comprehensive analysis of bioactives obtained from marine mollusks, crustaceans, and fish by-products, emphasizing modern extraction technologies with a focus on green and sustainable approaches. It further explores their mechanisms of action and documented efficacy in cosmetic formulations. Finally, the review outlines current limitations and offers future perspectives for the industrial valorization of marine by-products in functional and environmentally-conscious cosmetic development.

## 1. Introduction

Self-care has become a vital and significant part of our lives. This shift has led the beauty industry to seek new and innovative ideas, as well as novel elements for use in cosmetics and skincare products [1]. Increasingly, consumers are drawn to products designed to enhance and modify the appearance of skin, hair, and nails [2].

Over the past few decades, the highly successful cosmetics industry has turned its focus to the rich and mysterious world of marine resources, uncovering a wealth of information from the ocean’s depths [3]. Currently, multiple synthetic chemicals are used in cosmetic formulations, yet many fail to meet consumers’ expectations. As a result, there is growing interest in cosmetics that contain natural ingredients, as these are marketed as more sustainable, safer, and environmentally friendly [2].

Throughout history, natural products have played an important role in the treatment of chronic inflammation-related, skin, or other pestering, health-declining diseases and continue to be critical in the development of modern medicine [4]. Natural products (NPs) are characterized by a unique diversity of chemical structures, which enhances creativity in drug discovery. Traditionally, NPs have been sourced from terrestrial plants and bacteria. However, in the past 30 years, marine invertebrates, plants, and bacteria have also contributed significantly to the development of novel structural compounds, commonly referred to as marine natural products (MNPs) [1].

Given that approximately 70% of the Earth’s surface is covered by oceans—and that life originated there—the marine environment is a vast reservoir of biodiversity, consisting of around 500,000 species across roughly 28 phyla. Yet only nine types of marine animals and plants have been studied in significant detail for the isolation of natural products [5]. These include mollusks (such as sea hares and sea cucumbers), sponges, various algae (like green, blue-green, red, and brown species), phytoplankton (e.g., dinoflagellates), microorganisms (bacteria and fungi), coelenterates (like soft corals, gorgonians, and sea anemones), and bryozoans (e.g., *Bugula neritina*, which produces bryostatin compounds with notable anticancer and neuroprotective potential) [6,7,8].

Marine microorganisms, which are exposed to extreme environmental conditions—including high salinity, pressure, and low oxygen—can produce toxic chemicals either autonomously or by acquiring them from other marine organisms. These compounds may offer competitive advantages, help capture prey, or serve as chemical defenses against predators. As a result, the marine environment is largely viewed as a vast storehouse of novel bioactive natural products, many of which possess unique structural and chemical characteristics not found in terrestrial organisms. These bioactives are commonly categorized into groups such as terpenoids, sterols, polyethers, unsaturated fatty acids, alkaloids, glycosides, polysaccharides, peptides, and macrolides [5].

The first biologically active MNP was reported by Bergmann in the late 1950s [9]. Since then, marine ecosystems have been widely recognized for providing both direct and indirect benefits to humans. These ecosystems play vital roles in the biogeochemical cycling of elements, temperature regulation, and atmospheric gas control. Additionally, they serve as sources of nutrients, raw materials, and are essential for recreational and cultural activities [9].

Alongside a wide variety of animals, plants, and algae, the sea hosts numerous microorganisms, including bacteria, protozoa, and fungi. In the last decade, research has increasingly focused on the microscopic world, as the macroscopic marine world is relatively well-documented. Among marine microorganisms, fungi have historically received less scientific attention, but this is now rapidly changing [7,10]. Notably, marine fungi and microalgae are emerging as promising sources of novel bioactives with antioxidant, antimicrobial, and photoprotective potential. Microalgae such as *Haematococcus pluvialis* (rich in astaxanthin), *Chlorella*, and *Spirulina* are already being utilized in commercial skincare for their high content of pigments, fatty acids, and proteins. Marine fungi, historically understudied, are now being explored for secondary metabolites with cosmeceutical relevance [7,10].

Plant-derived ingredients are popular in cosmeceuticals, yet they present certain limitations, such as slow growth rates and variability in chemical composition depending on the region and season. In contrast, the ocean supports a vast range of fauna and flora biodiversity and offers a more chemically diverse selection of natural habitats. These marine resources can produce unique biomolecules, and, with the aid of modern aquaculture techniques, can be cultivated rapidly and cost-effectively [2,3]. In addition, advanced technologies such as genomics, proteomics, and metabolomics (the “omics” sciences) are increasingly used to accelerate the discovery of novel marine compounds and improve our understanding of their modes of action in human skin physiology. These approaches enable the targeted development of biofunctional ingredients and improve screening for safety and efficacy [11,12].

Several reviews have already examined the use of marine-derived bioactives with potential—and existing—applications in the cosmetic industry. This review aims to provide comprehensive information regarding the extraction techniques used to obtain bioactive compounds from marine sources. Additionally, it focuses on three major groups of marine invertebrates—mollusks, crustaceans, and fish—highlighting the bioactive compounds that can be derived from each. Finally, it explores the potential applications of these compounds in the cosmetic industry, analyzing their beneficial effects in skincare formulations and future perspectives.

### 1.1. Mollusks and Mollusk By-Products

The phylum Mollusca comprises a large group of invertebrate species that are referred to as marine mollusks, or more simply, mollusks [13]. While mollusks themselves are widely consumed as seafood, their by-products, often generated during seafood processing, represent valuable yet underutilized biomass for biotechnological and cosmetic valorization [13]. For example, these by-products include shells (calcium carbonate-rich) from oysters, mussels, and clams, which can be converted into calcium supplements, bioactive powders for toothpaste, and mineral fillers in cosmetics [14,15,16]. Moreover, mantle tissues and viscera (rich in peptides, amino acids, and enzymes), cephalopod ink (e.g., squid and cuttlefish, full of melanin), and chitin or chitosan are useful for cosmeceutical applications [17,18].

On Earth, this phylum is the second largest, as it represents numerous species both in terms of total number and diversity in morphology and ecological status [19]. With between 100,000 and 200,000 species, mollusks make up around 7% of all living animals, of which approximately 52,000 species are marine mollusks [20]. Oceans, seas, and estuaries are the marine environments where they are commonly found. Their inhabitants have adapted to a variety of life strategies, including trophic niches like predators, herbivores, scavengers, detritivores, filter feeders, and selected symbiotic photo- and chemo-autotrophs [20]. Mollusks have a soft body, which is usually covered by a hard shell, although not all species are protected by one. Snails, clams, squids, and octopuses are the most notable members of this phylum. The Mollusca phylum can be classified based on anatomical characteristics and ecological functions. Therefore, it can be divided into seven or eight main groups, each differing in features and adaptations [13,20].

Mollusks play a crucial role in biodiversity and ecosystem dynamics because they act as both predators and prey. The largest group is the gastropods, consisting of around 100,000 species, with body lengths ranging from 0.5 mm to 100 cm. They have various shell types, a freely movable head with eyes, a foot sole, and a helicoid visceral hump. The phenotypic expression of these features varies, with several exceptions [21]. In general, most gastropods have a single coiled shell layer, though some lack it entirely. They differ in their feeding habits and inhabit many marine environments [22]. Common representatives include limpets, snails, and sea slugs [13].

The second largest group in the Mollusca phylum is the bivalves, consisting of approximately 20,000 species and ranging in size from 1 mm to over 150 cm [21]. In contrast to gastropods, bivalves possess two shells connected via ligaments and muscles [22]. Examples include clams, oysters, geoducks, mussels, and scallops. Notably, bivalves are filter feeders, meaning they extract food particles from water using their gills [13,21].

The third largest group is the cephalopods, which includes approximately 30,000 species, with 1000 being marine and exhibiting body lengths ranging from 3 cm to 7 m [21]. Some species are regarded as the most intelligent and active invertebrates. They demonstrate complex behaviors, have an advanced nervous system, and are carnivorous [21,23]. Cephalopods are characterized by bilateral body symmetry, a prominent head, and strong arms or tentacles equipped with suckers. Examples include squids, nautiluses, cuttlefish, and octopuses [13,21].

Polyplacophora, also known as chitons, is another group within the Mollusca phylum. It includes around 1000 marine species with body lengths ranging from 3 mm to 30 cm [21]. These organisms have an oval-shaped body and differ due to their dorsal shell, which consists of eight overlapping plates or valves (Correia). Most species are herbivorous or detritivorous, feeding with the help of a strong rasping tongue called a radula. They are typically found in the intertidal zone [13,21]. Furthermore, Scaphopoda, commonly known as tusk-shells, is a small marine group comprising about 800 species. Their elongated, tubular shells resemble elephant tusks and range from 2 mm to 20 cm in length. They live buried in sediments, using a conical foot for burrowing and capturing foraminiferans with specialized tentacles called captacula [13,21].

The Monoplacophora class has very few extant members, with fewer than 30 known species, like *Tergomya*. These organisms have segmented bodies and a single cap-like shell. Though this class was once thought to be extinct, surviving specimens have been discovered in deep-sea habitats. These range in size from 1 to 40 mm and typically reside at depths between 200 and 7000 m. Their feeding habits resemble those of chitons, but they are distinguished by their singular dorsal shell [13,21]. Moreover, Aplacophorans are a lesser-known group of mollusks. These small, worm-like creatures either lack a shell or possess spicule-like body structures. They range from 1 to 3 mm in size, feed on small invertebrates, and are commonly found in deep-sea environments. They are classified into two subgroups: solenogasters and caudofoveates [13,21].

**Figure 1:**
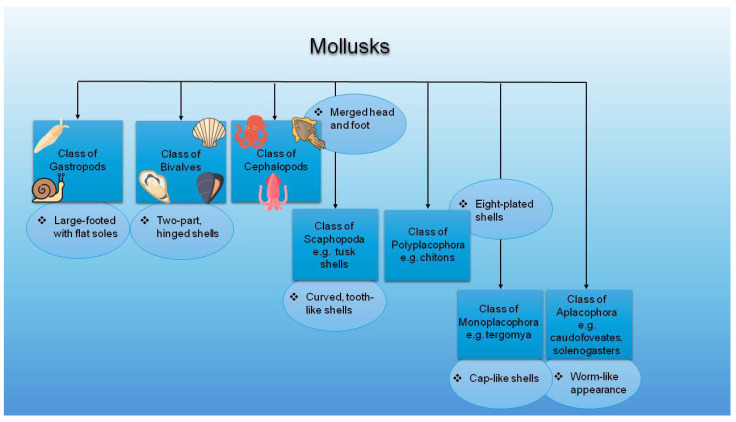
Classification of mollusks with examples (Figure designed with https://www.canva.com/ (accessed on 3 June 2025), and parts of this figure were obtained via https://www.flaticon.com/ (accessed on 3 June 2025)).

Gastropods, bivalves, and cephalopods are the largest groups within the Mollusca phylum. Despite differences, they share several characteristics. All three can inhabit freshwater environments and possess a foot, a shell, gills, and the ability to reproduce sexually. Additionally, they all feature a head and a radula. Importantly, all three groups possess a heart, although its anatomical complexity varies: gastropods have a single-chambered heart, bivalves possess a three-chambered heart, and cephalopods have a closed circulatory system with three hearts (two branchial, one systemic) [13,24,25,26]. Among these diverse molluscan classes, the gastropods, bivalves, and cephalopods are the most relevant to bioactive compound extraction and potential cosmetic applications [13]. For instance, certain mollusk-derived compounds, such as conchiolin proteins, glycosaminoglycans, taurine, and nacre extracts, are increasingly explored in skincare for their antioxidant and regenerative properties [27,28]. Figure 1 illustrates the classification of mollusks with specific examples in each group.

### 1.2. Crustaceans and Crustacean By-Products

Crustaceans (Crustacea) belong to the group of arthropods and are classified as the fourth-largest and most diverse animal group. They are considered a subphylum comprising approximately 50,000 to 75,000 species [29]. The term “Crustacea” is derived from the Latin word for shells and refers to the exoskeleton, which is not a true “shell” like those found in mollusks [30]. Examples of animals that belong in this group include crabs, lobsters, barnacles, woodlice, beach fleas, prawns, and a host of yet-undescribed species [29,31].

Large crustaceans breathe through gills, whereas smaller ones use their body surfaces. The majority of crustaceans are dioecious, meaning they have distinct male and female individuals. Reproduction processes vary among species. Crustaceans are an important food source for humans and also serve as a vital link in the oceanic food chain, acting as prey for animals such as whales, fish, and pinnipeds [32]. They can be found in both inland and oceanic waters, ranging from the Arctic to the Antarctic, and at elevations from 16,000 feet above sea level, including the Himalayas, to depths well below sea level.

The major groups of crustaceans can encompass Branchiopods (small, freshwater crustaceans, e.g., fairy shrimp, clam shrimp, tadpole shrimp, water fleas), Remipedia (blind, elongate crustaceans inhabiting anchialine caves (remipedes)), Cephalocarids (primitive benthic crustaceans that lack eyes, e.g., horseshoe shrimps), ostracods (small bivalved crustaceans in marine and freshwater environments (seed shrimps)), as well as Maxillopods and Malacostraca [30].

Maxillopods can be further divided into Copepoda (mainly planktonic or benthic organisms like sea louse, calanoida, or cyclops), Thecostraca (including sessile crustaceans with a hard, bivalved carapace such as facetatecta, ascothoracida, and barnacles), Branchiura (external parasites of fish (e.g., fish lice)), Mystacocarida (tiny worm-like interstitial organisms like mustache shrimp), Pentastomida (or tongue-worms, e.g., *Linguatula serrata*, *Armillifer*), Tantulocarida (microscopic, parasitic crustaceans like *Tantulacus dieteri*), and Cirripedia (sessile suspension feeders like goose or acorn barnacles) [30]. Malacostraca, on the contrary, can be subcategorized into three categories: Phyllocarida (leptostraca filter-feeding species), Hoplocarida (stomatopods (e.g., mantis shrimp)), and Eumalacostraca. Considering Eumalacostraca, they can be further divided into Eucarida (including large, active crustaceans decapods (e.g., crabs, lobsters, and shrimps), euphausiids (krill), and amphionides), Syncarida (freshwater or subterranean crustaceans (Bathynellacea and Anaspidacea species)), and Peracarida (involving diverse orders like Amphipods (e.g., sandhoppers)), Mysidacea (mysids, referred to as opossum shrimps), Thermosbaenacea (also known as cave microshrimps), Cumacea (also known as hooded or comma shrimps), Tanaidacea (known as tanaids), Speleogriphacea, and Isopods (land-based crustaceans like woodlice or pill bugs) [30,33].

Crustaceans are invertebrates with an open circulatory system (i.e., blood does not flow in a closed loop), a hard exoskeleton (carapace), a bilaterally symmetrical segmented body, and more than four pairs of jointed appendages (“legs”). The hard exoskeleton contains chitin, usually hardened with calcium carbonate, which protects them from predators and prevents water loss. They also possess a primitive ventral nerve cord and brain (ganglia near the antennae), eyes typically located on stalks, a straight digestive tract with grinding structures, and two digestive glands. They expel waste through a pair of green glands and breathe using gills located near the base of the antennae [34].

A crustacean’s body is typically divided into three parts: the head (cephalon), thorax, and abdomen (pleon). In some species, the head and thorax are fused into a cephalothorax, which is covered by a single large carapace. All three body regions may bear biramous, jointed appendages. All species possess two pairs of maxillae, two pairs of antennae, and mandibles during at least one life stage. The presence of two pairs of antennae distinguishes crustaceans from insects, one of the other main arthropod groups. The cephalic region is composed of six basic appendages: compound eyes, first antennae (biramous in malacostracans), second antennae, mandibles, first maxillae, and second maxillae. The two antennae pairs serve mainly sensory roles, such as food detection and filtering, whereas the last three pairs are involved in acquiring, manipulating, and processing food [30]. In contrast, the thorax and abdomen differ in the number and type of appendages depending on the taxonomic group. For example, malacostracans—such as decapods and amphipods—usually possess five to eight pairs of thoracic appendages (called thoracopods or pereiopods), and six pairs of abdominal appendages (pleopods and terminal uropods) [34].

The circulatory system in crustaceans is open, and blood is directed toward the heart, which lies near the dorsal side. Hemocyanin is the copper-binding, oxygen-carrying protein pigment in Malacostraca, whereas hemoglobin is found in copepods, ostracods, barnacles, and brachiopods. The alimentary canal typically includes a “gastric mill” that functions like a gizzard, along with two digestive glands responsible for nutrient absorption. Other internal structures encompass kidney-like organs and a brain (ganglia) located near the antennae, as well as a central cluster of ganglia beneath the gut [34].

Crustaceans undergo molting as they grow since their exoskeletons do not expand. The molting process can last from a few minutes to several hours. During molting, a new soft exoskeleton develops underneath the old one, which is then shed. Crustaceans are vulnerable during this time until the new exoskeleton hardens. They typically increase their body size by 40–80% almost immediately after molting. The majority of crustaceans reproduce sexually through distinct male and female individuals [32]. At this point, it must be noted that crustaceans are also classified based on body size. Small-sized crustaceans belong to the group Entomostraca, while larger ones belong to the Malacostraca. Entomostraca is not a valid taxonomic division, as its members differ significantly. In contrast, Malacostraca is a well-defined and valid division characterized by features such as abdominal appendages, a gastric mill, an eight-segmented thorax, and an abdomen composed of six (sometimes seven or eight) segments [31].

**Figure 2:**
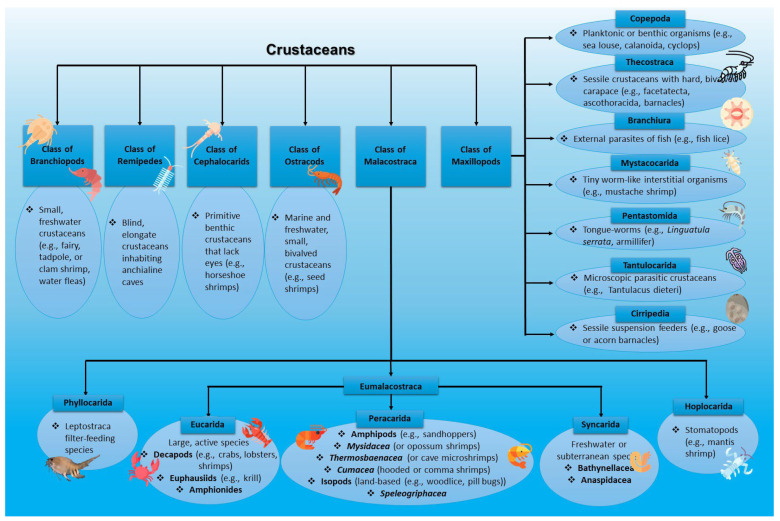
Classification of crustaceans with examples (Figure designed with https://www.canva.com/ (accessed on 20 June 2025) and parts of this figure were obtained via https://www.flaticon.com/ (accessed on 20 June 2025)).

From a cosmeceutical perspective, crustacean by-products such as shells and exoskeletons are valuable sources of chitin and its derivative chitosan, which are widely used in skincare products for their biocompatibility, moisturizing, wound-healing, and antimicrobial properties. Additionally, crustaceans such as krill and shrimp are rich in astaxanthin, a potent antioxidant known to protect the skin from oxidative stress and ultraviolet (UV)-induced damage [35,36]. A comprehensive classification of crustaceans is depicted in Figure 2.

### 1.3. Fish and Fish By-Products

Fish are a rich source of high-quality protein and are consumed either in fresh or processed form. The skin, bones, scales, heads, and viscera are among the various components of the marine biomass that account for approximately 50 to 70% of total fish weight and are typically discarded during processing. These components are regarded as secondary raw materials with high valorization potential. Bioactive compounds obtained from fish and their by-products can be used in various industries, including food, agriculture, cosmetics, nutraceuticals, and pharmaceuticals [37].

Fishery discards—comprising both fishery bycatch and processing by-products—represent a growing environmental and economic concern [37]. These discards are difficult to handle and must be managed as certified by-products due to their high content of polyunsaturated lipids and endogenous proteolytic enzymes, which accelerate degradation. Moreover, the cost of proper disposal places a financial burden on fisheries and fish processing industries. Therefore, utilizing fishery waste as a secondary raw material is increasingly considered a sustainable and resource-efficient solution. This approach minimizes environmental impact, promotes circular economy practices, and contributes to the long-term sustainability of the fishing sector [38,39].

It has been acknowledged that marine species and their by-products are a remarkable source of novel bioactive metabolites. Among marine vertebrates, a wide range of fish species contribute to the generation of biomass by-products. These include both fatty and lean species such as salmon, mackerel, sardine, herring, cod, and tuna. Each species offers different types of bioactive molecules based on fat content, tissue type, and seasonal variation. For example, oily fish such as salmon and mackerel are rich in ω-3 fatty acids, while lean fish (e.g., cod) are excellent sources of high-purity collagen and gelatin [40,41]. Although this review focuses on bioactive use in cosmetics, it is worth noting that fish by-products are also widely used in animal feed, fertilizers, and biomedical applications. However, recently, scientific focus has shifted toward the identification and isolation of high-value bioactives with skin-protective, anti-aging, and antioxidant effects [40,41].

Fish waste contains a wide range of valuable compounds, such as proteins and peptides (including collagen, gelatin, myosin, actin), essential fatty acids (e.g., eicosapentaenoic acid (EPA) and docosahexaenoic acid (DHA) ω-3 fatty acids), vitamins (A, D, and E) and minerals (e.g., calcium, zinc, selenium), enzymes (e.g., proteases, lipases), and pigments and carotenoids (e.g., astaxanthin, especially in salmon skin). These compounds are being increasingly recognized for their therapeutic, nutritional, and cosmeceutical applications [42,43,44,45].

Fish-derived collagen and collagen peptides are among the most widely applied ingredients in anti-aging creams, hydrating gels, facial masks, and wound-healing formulations due to their biocompatibility and excellent skin regeneration properties. Fish oil rich in ω-3 fatty acids is also utilized in skincare products for its anti-inflammatory and barrier-restoring effects. Furthermore, antioxidant-rich fish protein hydrolysates and bioactive peptides are increasingly studied for their photoprotective, anti-wrinkle, and anti-pigmentation potential [46,47]. Table 1 represents the challenges and benefits in the valorization of crustacean by-products.

## 2. Extraction Techniques of Bioactive Compounds

Extraction techniques are generally classified based on the mechanism employed: thermal (e.g., microwave-assisted extraction (MAE), subcritical water extraction (SWE)), mechanical (e.g., ultrasound-assisted extraction (UAE)), solvent-based (e.g., Soxhlet, supercritical fluid extraction (SFE), or biochemical (e.g., enzyme-assisted extraction (EAE)). The selection of the appropriate method depends on the nature of the source material, target bioactive compound, and intended industrial application [37].

### 2.1. Conventional Extraction Techniques

Conventional, otherwise known as traditional, extraction techniques are the most frequently used approaches for isolating bioactive compounds. These include the solid–liquid extractions that can be performed in different ways, such as boiling the sample with the solvent with or without stirring for a specified duration, refluxing using a Soxhlet apparatus, percolation, or maceration with constant agitation. Depending on the target molecules, various solvents are employed in large quantities, including water, methanol, ethanol, acetonitrile, ethyl acetate, acetone, and dichloromethane [50].

A significant disadvantage of traditional techniques is that they are labor-intensive and time-consuming, relying heavily on solvent diffusion and prolonged extraction times. They can also consume large volumes of solvents, which may lead to sample contamination and generate hazardous solvent waste, posing risks to both health and the environment [51]. The Soxhlet, in particular, involves elevated temperatures, which increase the risk of thermal degradation of heat-sensitive (thermolabile) compounds [52]. Additionally, scaling up these technologies to an industrial level is challenging due to economic, practical, environmental, and energy-related limitations [53].

To overcome the limitations of traditional techniques, several modern extraction methods have been developed [51].

### 2.2. Non-Conventional Extraction Techniques

Numerous alternative methods have been developed in response to the growing demand for eco-friendly, efficient, and economical extraction processes. These non-traditional techniques aim to address the drawbacks of conventional methods and include pressurized liquid extraction (PLE), SWE, supercritical fluid extraction (SC-CO_2_), EAE, MAE, and UAE [51,52,53]. These techniques will be further analyzed below. Their primary advantages include higher extraction yields, improved recovery quality of bioactives, shorter processing times, nontoxic solvents, and greater cost-effectiveness [51].

#### 2.2.1. Enzyme-Assisted Extraction (EAE)

EAE is based on a catalytic hydrolysis, where enzymes disrupt the cell wall structure to release intracellular components into the extraction medium [54]. Key parameters influencing the efficiency of the process are multiple, including temperature, pH, proportion of substrate-to-enzyme ratio, solvent type, and agitation speed.

This technique is a relatively new approach for marine biomaterial extraction and is still under active research to optimize conditions and identify the most suitable enzymes [13]. Food-grade enzymes such as proteases and carbohydrates are commonly used to break down cellular matrices and facilitate compound release, particularly important for marine algae, whose cuticles are composed of complex, heterogeneous biomolecules [54].

Compared to traditional methods, EAE offers several benefits like lower operating temperatures, the use of environmentally-friendly solvents and nontoxic solvents, higher extraction yields, economic feasibility due to food-grade enzyme availability, and the transformation of water-insoluble materials into soluble ones [13,51,54].

EAE is well-suited for the recovery of thermolabile or protein-based compounds, like collagen and peptides from fish and mollusk tissues, as well as polysaccharides from marine algae. The main limitations involve enzyme cost, substrate specificity, and the need for downstream purification of the hydrolysates [37,55].

#### 2.2.2. Microwave-Assisted Extraction (MAE)

MAE uses microwave radiation to extract a variety of bioactive compounds from natural sources. This technique is considered simple, requiring relatively few reagents and only small amounts of organic solvents [13].

Microwaves operate at frequencies ranging from 300 MHz and 300 GHz (wavelengths of 1 mm and 1 m). This oscillating electromagnetic field causes polar molecules to rotate and create internal friction, which produces localized heating [56]. The resulting thermal pressure ruptures cells, releasing their contents into the surrounding solvent [13,57].

Polar solvents such as ethanol and methanol are preferred in MAE compared to non-polar ones, due to their higher dielectric constants, allowing rapid heating and enhanced extraction efficiency [13]. In contrast, non-polar solvents such as hexane and chloroform are less effective for microwave energy absorption [56].

MAE is ideal for extracting polyphenols, essential oils, and pigments from marine algae, crustacean shells, and fish skin. A challenge in MAE is the uneven heating in bulk-scale systems, which may lead to degradation or inconsistent yields in industrial applications [37,58].

#### 2.2.3. Subcritical Water Extraction (SWE)

SWE is a green extraction method that enables efficient compound recovery in short periods with minimal use of organic solvents [13,59]. In marine algae, this method has shown extraction efficiencies of up to 4.9% [60].

The mechanism follows the “like dissolves like” principle, where polar solvents extract polar compounds and non-polar solvents extract non-polar ones [60,61]. In SWE, water is kept in its liquid state at elevated pressure and temperature [13]. The dielectric constant of water decreases with increasing temperature, enabling the selective extraction of either polar or non-polar compounds based on the temperature setting. High temperatures are required for extracting large or complex molecules [60]. SWE differs from traditional extraction due to water’s high dielectric strength, polarity modulation capacity, and high boiling point relative to mass [13].

SWE has shown promising results in the extraction of polysaccharides, phenolic acids, and antioxidant pigments from algae and fish skin. The high-pressure requirement and material corrosion risk are important engineering challenges for SWE scale-up [37,60].

#### 2.2.4. Supercritical Fluid Extraction (SFE)

SFE is considered one of the most environmentally friendly modern extraction methods [62]. It involves extracting compounds using fluids in their supercritical state, where they exhibit properties of both gases and liquids. The process consists of two key steps: extraction with a supercritical fluid and separation of the analyte from the fluid [63].

Raw materials are loaded into an extractor vessel, which is then pressurized and heated above the fluid’s critical point. A pump delivers the fluid, which dissolves the analytes and carries them into a separator where the compounds are collected. Common supercritical fluids, including fluorocarbons, CO_2_, ethylene, methane, nitrogen, and xenon, can be used as solvents in this extraction method [64]. Generally, supercritical fluids have a high diffusion coefficient and low viscosity, which allows them to readily enter sample particles and carry the soluble analytes with them. Carbon dioxide (CO_2_) is the most widely used supercritical fluid because it is non-polar, nontoxic, inexpensive, largely inert, non-flammable, and recyclable [63]. Moreover, CO_2_ operates under a favorable critical temperature (Tc) of 1 °C and pressure (Pc) of 74.8 atm. To improve the solubility of polar compounds, CO_2_ is often combined with modifiers like ethanol or methanol [65]. Plus, pharmaceutically important chemicals can be extracted via supercritical carbon dioxide (SC-CO_2_) extraction [64].

Advantages of SFE include lower solvent usage, faster extraction, compatibility with automated systems, high purity of extracts, high selectivity, and potential for integration with analytical techniques (e.g., SFE/gas chromatography (GC), SFE/high performance liquid chromatography (HPLC)) [65].

SFE is especially effective for the selective recovery of lipophilic compounds, such as ω-3 fatty acids, astaxanthin, or squalene from marine oils and crustacean shells [37,66]. Recent innovations involve coupling SFE with in-line fractionation of membrane filtration to increase purity and reduce post-processing [67].

#### 2.2.5. Ultrasound-Assisted Extraction (UAE)

UAE, also referred to as “clean technology”, is an innovative extraction technique gaining popularity due to its many benefits over conventional methods. These include low solvent volumes, short extraction times, minimal equipment, and reduced environmental and economic impacts. UAE employs ultrasonic waves with frequencies between 20 kHz and 10 MHz [68,69,70]. Two main ranges are distinguished: (i) high-power ultrasound (20–100 kHz), used for extraction and processing; and (ii) signal or diagnostic ultrasound (100 kHz–10 MHz), used for quality control and assessment, as well as clinical imaging [71]. Ultrasound generates cavitation bubbles that implode and create localized mechanical effects, facilitating the rupture of cell walls and enhancing solvent penetration into tissues, thereby increasing extraction efficiency. UAE is therefore highly suitable for the extraction of proteins, pigments, and small peptides from fishery waste, seaweeds, and crustaceans, while preserving compound integrity [37,57].

Nevertheless, such modern extraction techniques, which can increase the yield on a lab scale, cannot be put into daily industrial practice due to a lack of instrumentation and infrastructure, as well as experienced personnel to cover the extraction of the bulk amounts of daily produced marine by-products. Therefore, using nontoxic green solvents for classic green extraction approaches via blending the solid by-products with solvents and then filtering the extract from the remnants, is still the most applicable method for upscaling extraction procedures for daily use in huge infrastructures. Green solvents can be recycled through flash rotary evaporators and be reused. However, large volumes of organic solvents, despite being green, increases the safety measures needed for daily use industrially. In recent years, hybrid combinations such as UAE-EAE or UAE-SFE have been explored to enhance yields, reduce time, and minimize energy consumption [57]. The benefits and challenges of the most frequently used extraction methods are presented in Table 2.

## 3. Bioactive Compounds from Marine By-Products

**Figure 3:**
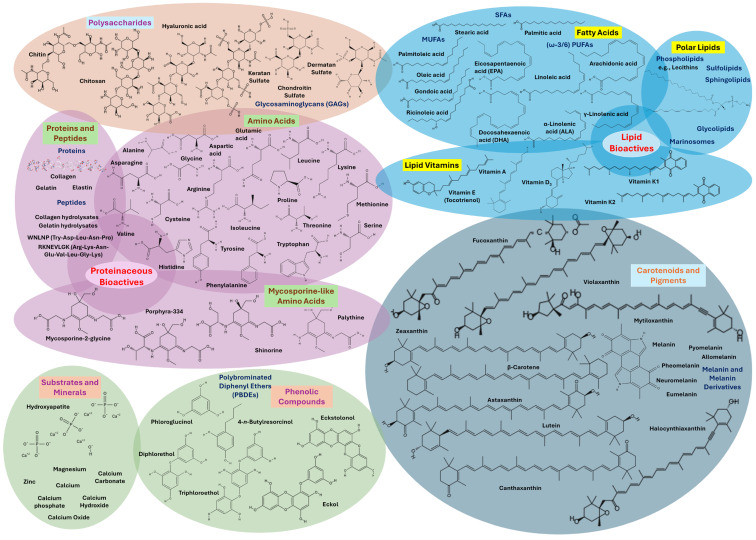
Bioactive compounds in marine bycatch and by-products. (Parts of this figure were obtained from https://molview.org/ (accessed on 22 June 2025).).

Marine by-products such as fish heads, skins, bones, viscera, crustacean shells, and mollusk tissues are abundant in valuable bioactive compounds, like polysaccharides (e.g., chitin, chitosan, and their derivatives, hyaluronic acid, GAGs, and fucoidans), fatty acids (e.g., SFAs, MUFAs, and mainly PUFAs like LA, DHA, EPA, ALA, or arachidonic acid), lipid bioactives and polar lipids (e.g., lecithin, phospholipids, glycolipids, sphingolipids, sulfolipids, or marinosomes), lipid vitamins (e.g., A, B, D, E, or K), amino acids (all 20 standard residues and mostly MAAs), proteins (e.g., collagen, elastin, gelatin), peptides (small amino acid chains, collagen- and gelatin-hydrolysates), carotenoids and pigments (e.g., astaxanthin, melanins like allomelanin, neuromelanin, eumelanin, pheomelanin, and pyomelanin, lutein, β-carotene, halocynthiaxanthin, and fucoxanthin), phenolic compounds (e.g., polyphenols, PBDEs, phlorotannins like diphlorethol, triphloroethol, trifuhalol, and tetrafuhalol, phloroglucinol, eckol, and eckstolonol), substrates and minerals (e.g., hydroxyapatite, CaPs, minerals like Ca, Mg, and Zn, and powdered pearl shells or nacreous shell layers). These compounds exhibit a broad spectrum of biological activities, such as antioxidant, anti-inflammatory, antimicrobial, anti-aging, moisturizing, and UV-protective properties, making them ideal for cosmetic use. Their extraction not only supports the development of functional skincare products but also contributes to environmental sustainability by promoting the circular use of marine resources [11,28,78,79]. Figure 3 illustrates the bioactives in marine bycatch and by-products.

### 3.1. Bioactive Compounds and Structural Components of Mollusks

#### 3.1.1. Shell Structure of Mollusks

The typical structure of a molluscan shell, such as those of oysters, mussels, abalone, and nautilus shells, is composed of three layers [80]. These include the periostracum and two calcified layers. The periostracum (outermost layer) is a thin organic leathery material. The two calcified layers are the prismatic (outer) and the nacreous (inner) layers [81].

The prismatic layer is made up of long calcitic crystals arranged perpendicularly to the periostracum. The nacreous layer, also known as mother-of-pearl, consists of aragonite crystals organized in a laminar, brick-wall-like pattern [82]. Nacre is particularly valued for its high fracture-resistance properties [83]. Shell formation involves secretion by the mantle epithelium of an organic matrix composed of proteins, peptides, lipids, and carbohydrates, along with the inorganic mineral calcium carbonate [84]. Specifically, the shell matrix includes glycoproteins, chitin, lipids, acidic polysaccharides, and structural proteins [85].

#### 3.1.2. Terpenes and Other Bioactive Metabolites in Mollusks

Mollusks are one of the most chemically diverse invertebrate groups, particularly rich in terpenes. Terpenoids, also known as isoprenoids, represent the largest class of natural products, and this vast pool of complexity allows for a wide range of interactions with biological targets. These compounds are often multicyclic, oxygen-containing molecules with broad biological activity [86].

Marine and estuarine mollusks produce a wide variety of terpenoid metabolites, like chamigrene, amphilectane, and cembrane derivatives. Many of these molecules feature rare groups such as isothiocyanates, dichloroimines, isonitriles, isocyanates, and halogens. Terpene biosynthesis in mollusks can occur de novo, starting from mevalonic acid and leading to the production of sesterterpenes. In nudibranchs (gastropods, Opisthobranchia), small molecules are used for functions including communication, reproduction, and predator defense [20,87].

#### 3.1.3. Cephalopods: Protein-Rich Tissues and Bioactive Ink

Cephalopods are rich in proteins and peptides, including high levels of collagen and mucins [88]. Mucins, the main component of mucus, help maintain homeostasis and prevent desiccation [89]. Other identified peptides include hemocyanin fragments and various muscular proteins such as myosin, paramyosin, and tropomyosin, often attributed to muscle contamination during sampling. Even though respiration is mainly performed via the gills, the presence of hemocyanin in the mucus may indicate a possible gas exchange [88,90].

Cephalopod ink, mainly produced by squids and octopuses, is a rich source of bioactive compounds. These include melanin, tyrosinase, catecholamines, amino acids, and metals. Melanin is a naturally occurring pigment in organisms like bacteria, fungi, plants, and animals and has multiple roles, as it originates from amino acids, but is not a protein. Therefore, it is a complex biopolymer present in two forms: eumelanin and pheomelanin, which differ in their molecular precursor [91]. Eumelanin is a polymer of tyrosinase derived from 5,6-dihydroxyindole (DHI) and 5,6-dihydroxyindole-2-carboxylic acid (DHICA), compared to pheomelanin, which is made up when benzothiazine and benzothiazole monomers produced when cysteine is present. Also, eumelanin has a dark brown color, whereas pheomelanin presents as an orange-red compound [92,93].

Tyrosinase, additionally, is the precursor used in melanin production; thus, it is a key enzyme in melanin biosynthesis, primarily found in cephalopods’ ink [91]. Furthermore, low nanomolar to low micromolar amounts of catecholamines, including dihydroxyphenylalanine (DOPA) and dopamine (monoamines), are also reported to be present in the ink [94,95,96]. Free amino acids, especially taurine and glutamate, as well as lysine, leucine, and arginine, are highly concentrated in cephalopod ink. These contribute to chemical defense through phagomimetic properties [97,98,99]. Plus, regarding metals, high levels of lead, copper, and cadmium are present in the cephalopod ink [100], potentially acting as enzyme cofactors in melanin synthesis [99].

#### 3.1.4. Gastropods and Bivalves: Minerals and Metabolites

Gastropods are an abundant source of minerals such as potassium, iron, zinc, selenium, sodium, and sulfur [101]. Notably, they have higher values of iron and selenium compared to other mollusks [102]. Their metabolite composition includes ~3% sterols and ~55% terpenes, while bivalves contain ~41% sterols and ~5% terpenes. The metabolic alterations in the reproductive pathways of bivalve mollusks may be the cause of their higher sterol content. Mollusks also exhibit higher levels of alkaloids (5–6%) and polyproprionates (13–32%), though nitrogenous compounds are less common [20].

Bivalves are an affordable source of proteins, minerals, amino acids, and vitamins, including E and D resolvins that contribute to prostanoid synthesis [103]. They contain zinc (226 ± 560 mg/kg) and copper (41 ± 110 mg/kg), along with moderate iron and selenium [102], and are especially rich in vitamin D and E (tocotrienol) [104,105]. Carotenoids like astaxanthin, canthaxanthin, mytiloxanthin, zeaxanthin, and their esters are also abundant [106].

#### 3.1.5. Oysters: Peptides, Minerals, and Shell Components

Oysters, a type of bivalve mollusk, are rich in bioactive peptides. Oyster proteins, when hydrolyzed, yield bioactive peptides with antioxidant, antimicrobial, and other therapeutic properties. Due to their stability and variety of biological activities, oyster protein hydrolysates (OPHs) and peptides (OPs) have recently gathered attention [107,108,109].

Oyster soft tissues are particularly rich in zinc [110]. Oyster shells are primarily composed of calcium carbonate (CaCO_3_) (~95%) and a small percentage of organic shell (skeleton) matrix proteins (~0.1–5%) [111]. Other calcium compounds like calcium oxide (CaO) and calcium hydroxide (Ca(OH)_2_) may be derived through processing. Calcinated oyster shell powder has especially attracted attention for its antibacterial and biocidal properties, as well as its biocompatibility [112]. Other components of the shell include polysaccharides (mainly chitin), soluble and insoluble proteins (e.g., glycoproteins, collagen, gelatin, albumins), lipids, free amino acids, short peptides, and pigments [83].

Oyster peptides, found in both tissues and mantle, are highly valued for their antioxidant and immunomodulatory effects. Antimicrobial peptides (AMPs) are abundant in oyster blood cells, gills, and mantle tissue, and are an active area of research for natural marine therapeutics [112].

### 3.2. Bioactive Compounds and Structural Components of Crustaceans

#### 3.2.1. Shell Structure of Crustaceans

**Figure 4:**
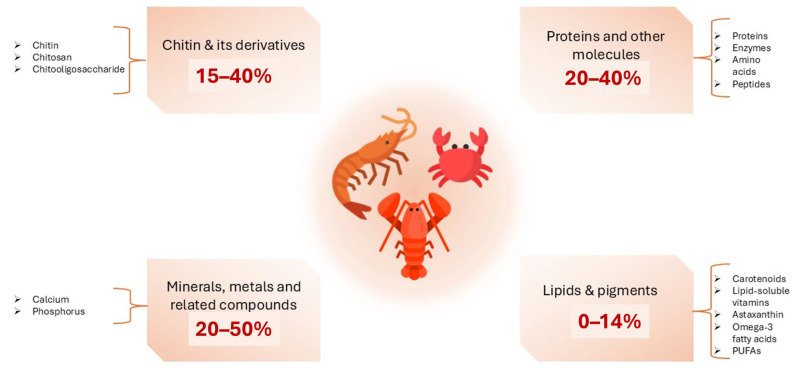
Bioactive compounds in crustaceans (Figure designed with https://www.canva.com/ (accessed on 16 July 2025), and parts of this figure were obtained via https://www.flaticon.com/ (accessed on 16 July 2025)).

Crustacean shells are composed of chitin (15–40%), protein (20–40%), calcium carbonate (20–50%), and lipids (0–14%) along with high concentrations of ω-3 fatty acids, pigments, and other minor components. The composition may vary depending on season and species [34,113,114]. These components have demonstrated a wide range of uses. For example, calcium carbonate can be applied as fertilizer, filler, or white pigment, whereas proteins can be utilized as animal feed or fertilizer [115]. Chitin, on the other hand, has versatile applications, ranging from water treatment to biomaterials, functional food, medicine, and cosmetics (Figure 4) [116,117].

#### 3.2.2. Chitin and Its Derivatives

Chitin (C_8_H_13_O_5_N) is the second most prevalent polysaccharide in nature after cellulose. It is made of (1→4)-N-acetyl-d-glucosamine monomers [118,119] and is structurally similar to cellulose, except for the presence of 2-acetamido-2-deoxy-β-d-glucose (NAG) units, joined by β(1→4) linkages [118]. Chitin is also a white or yellowish, highly hydrophobic, tasteless, and odorless substance [120]. Its crystalline structure is stabilized by intramolecular hydrogen bonds, making it insoluble in water and many organic solvents [121]. Only a few solvents, like fluorinated solvents (hexafluoropropanol, hexafluoroacetone), chloroalcohol–mineral acid mixtures, and dimethylacetamide with 5% lithium chloride, can dissolve it [122].

Chitosan is produced via deacetylation of chitin through enzymatic or chemical hydrolysis [123]. It is composed of d-glucosamine (deacetylated) and N-acetyl-d-glucosamine (acetylated) copolymer units linked via β (1,4) bonds [122]. Simply, in the -NH_2_ group of the d-glucosamine unit that repeats, a proton is added in contrast to the same medium where the polysaccharide is converted into a polyelectrolyte [124]. Chitosan is a cationic, acid-soluble heteropolymer found in crustaceans, mollusks, arthropods, and insects [121,125]. Its physicochemical properties and applications depend largely on its degree of acetylation [121].

Hydrolysis of chitin or chitosan results in chitooligosaccharides (COS). A variety of physical techniques, including hydrothermal, microwave, ultrasound, chemical, acidic, and enzymatic approaches, can be applied to depolymerize and generate COS [122,126]. COS are short-chained, low molecular weight, water-soluble, and low viscosity molecules with biological activities such as antibacterial, anticancer, cholesterol-lowering, and immunostimulant effects [127,128].

The term “chitin” originates from the Greek word “chiton”, meaning “a coat of mail” [119]. In nature, chitin is arranged in microfibrils and is one of the main components found in the exoskeleton of marine animals like crustaceans and insects, in the cell wall of fungi, as well as in octopuses’ beaks [118,127,129]. Hatchett first extracted chitin in 1799 using mineral acids from prawns, crayfish, and mollusk shells [130]. Following demineralization and deproteinization, chitin is extracted from the exoskeleton of crustaceans. Despite its insolubility, derivatives such as chitosan have enabled widespread use in medicine, food, agriculture, and cosmetics due to their biocompatibility, biodegradability, nontoxicity, recyclability, and bioactivity and their exceptional properties including their anticancer and antitumor [127,131,132,133,134], antimicrobial [135,136,137], antioxidant [138,139], anti-hypertensive [140], anti-inflammatory [141], anti-diabetic [142,143], hypocholesterolemic [144,145], and anti-coagulant [146] effects.

#### 3.2.3. Lipids and Pigments

Crustacean shells are rich in ω-3 polyunsaturated fatty acids (PUFAs), especially EPA and DHA, along with lipid-soluble vitamins (A, D, E, and K), while recently, anti-inflammatory bioactive polar lipids were also found in some species [36,147]. However, the lipid content varies by species, sex, climate, and environmental conditions [148,149]. These lipids are most concentrated in organs like the liver and pancreas of the shrimp, the head of the lobster, and the gonads of the crabs. According to Albalat et al., lobsters typically exhibit the highest lipid content compared to shrimps and crabs [150]. Another study where the lipid content between shrimp and crabs was compared showed that crabs have a significantly smaller amount of lipids in edible tissues than shrimps [151].

Tsoupras et al. [147] have found that shrimp extracts rich in amphiphilic compounds are rich in marine phenolics, carotenoids, polar lipids, and unsaturated fatty acids (UFAs), and they exhibited strong antioxidant capacity due to their phenolics and carotenoids contents, while most importantly they also showed potent anti-inflammatory and antithrombotic activities. These activities were attributed to the UFAs content of the shrimp polar lipids detected in these extracts, such as the monounsaturated fatty acid (MUFA) oleic acid (C18:1n9) and especially the omega-3 (n3) polyunsaturated fatty acids (PUFAs) like eicosapentaenoic acid (EPA; C20:5n3) and docosahexaenoic acid (DHA; C22:6n3), with favorable anti-inflammatory values for their n6/n3 PUFA ratio. Shrimp amphiphilic bioactives, like those rich in UFA polar lipids, provide anti-inflammatory effects against the platelet-activating factor (PAF) inflammatory pathway and antithrombotic effects against the ADP and eicosanoid thrombotic pathways of platelet activation. Such findings support further study on the use of shrimp extracts rich in anti-inflammatory, anti-thrombotic, and antioxidant amphiphilic bioactives as ingredients to produce new biofunctional health-promoting products, in the context of sustainable development and circular economy [147].

Moreover, the presence of carotenoids in such marine organisms is also of great importance. Carotenoids are lipid-soluble pigments (red, orange, and yellow) found in both photosynthetic and non-photosynthetic bacteria and fungi, with well-known antioxidant properties. They are responsible for the color, tints, and tones of many fruits (e.g., tomato, mango), vegetables (e.g., broccoli, spinach), and other plant-derived products, mainly due to their long polyenic carbon chain [113,122,152]. Recent data support that over 1200 natural carotenoids have been found in 722 source organisms [153]. Structurally, over 95% of carotenoids are made of isoprene units (C5 blocks) and are classified as C40 (8 isoprenoid units), C45 (9 isoprenoid units), C50 (10 isoprenoid units), and C60 (6 isoprenoid units). Since they are produced by bacteria, archaea, and eukaryotic species, C40 carotenoids are the most prevalent. However, both bacteria and archaea can synthesize C30 and C50 carotenoids, but only certain bacteria can synthesize C45 ones. Reports also indicate the presence of some apocarotenoids with typical C40 backbones (shortened from one or both ends), crocetin (C20), and bixin (C25). In certain situations, vitamin A is also regarded as an apocarotenoid, since β-carotene symmetrically cleaves to produce two identical retinal molecules [154,155,156].

Based on their structure, carotenoids can be categorized into two big groups, namely carotenes and xanthophylls. Carotenes include linear hydrocarbons, mostly with a cyclic molecule at the end, such as β-carotene, lycopene, while xanthophylls, derivatives of carotenes, contain one or more oxygenated carotenoids (oxygen in epoxy, methoxy, keto, and hydroxy groups). Marine-sourced xanthophylls include astaxanthin, zeaxanthin, violaxanthin, cryptoxanthin, and capsanthin [113,154,155,156].

##### Astaxanthin

Astaxanthin (C_40_H_52_O_4_, 3,3-dihydroxy-β, β-carotene-4,4-dione) is a widely accepted marine carotenoid [113]. It is a red-orange, fat-soluble xanthophyll, structurally characterized as a ketocarotenoid, as it has a hydroxyl (OH) and a keto (CO) group at both ends and potent antioxidant properties [122,149,157,158]. Moreover, the presence of two chiral carbons at positions 3 and 3′ in its structure enables the formation of many isomers and stereoisomers [159,160,161]. It exists in *cis/trans* geometrical (*E* and *Z*) and stereoisomeric forms (e.g., (3*S*,3′*S*; 3*R*,3′*R*) [162,163,164], often esterified with fatty acids or bound to proteins (carotenoproteins).

**Figure 5:**
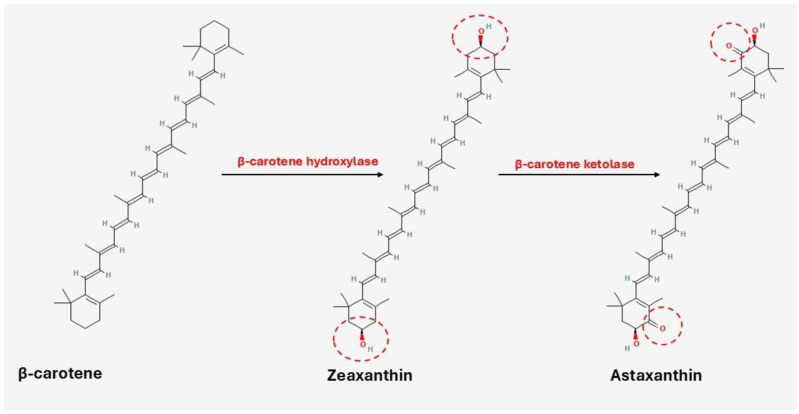
Conversion of β-carotene and zeaxanthin into astaxanthin (Parts of this figure were obtained from https://molview.org/ (accessed on 3 June 2025)).

Astaxanthin is the amphiphilic by-product of β-carotene and zeaxanthin with the help of β-carotene hydroxylase and β-carotene ketolase, respectively, as shown in Figure 5 [165,166,167]. It demonstrates superior antioxidant activity: 10× higher than zeaxanthin, lutein, β-carotene, and canthaxanthin and 100× higher than α-tocopherol [148]. Furthermore, it exists in unesterified (free) and esterified (mono- and di-esterified) derivatives contributing to its bioactivity and stability [113,149]. The free form is unstable and may undergo degradation and oxidation when exposed to light, prolonged heat, oxygen, and extreme pH conditions. Astaxanthin’s hydroxyl groups esterify with many fatty acids, including linoleic, palmitic, oleic, and stearic, while they may combine with carotenoproteins or carotenolipoproteins to produce complexes (e.g., in crustaceans) [122,149,168].

#### 3.2.4. Proteins and Protein Hydrolysates

Protein hydrolysates constitute about 40% of crustacean by-products. They include amino acids (e.g., arginine, glycine), peptides, oligopeptides, and non-protein nitrogen sources (e.g., nucleotides), acting as valuable nutrients [122,148,169]. Hydrolysates produced via acid, alkaline, or enzymatic hydrolysis have improved solubility, emulsification, and bioactivity [169,170]. Protein content varies depending on the species, environment, season, reproductive condition, and processing method used [169], while peptides of different sizes can provide free amino acids [122]. Moreover, shrimps generally contain more protein than crabs [169].

#### 3.2.5. Minerals and Metals

Crustacean shells contain essential minerals including phosphorus, magnesium, calcium, and nitrogen [148]. Their concentrations depend on the species, extraction, and demineralization method [171,172]. Using a variety of acids, different calcium salts can be produced under specific temperature, time, and acid concentration conditions. Calcium carbonate is the most abundant, occurring as calcite, amorphous calcium carbonate, or tricalcium phosphate (hydroxyapatite) [173].

### 3.3. Fish Bioactive Compounds: Composition, Types, and Applications

**Figure 6:**
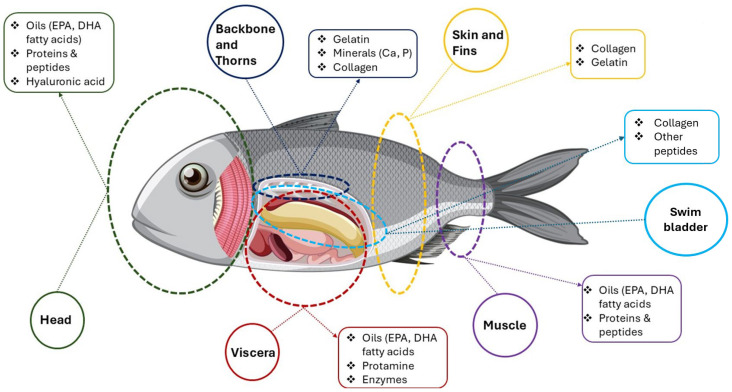
Fish parts and bioactive compounds extracted from them (Parts of this figure were obtained from https://www.freepik.com (accessed on 3 June 2025)).

Figure 6 is a schematic diagram illustrating the valorization of different fish by-products, highlighting the valuable bioactive compounds that can be extracted from various parts of a fish. It essentially shows how different anatomical sections, often considered waste from fish processing, can be utilized to obtain beneficial components for various applications (e.g., functional foods, nutraceuticals, cosmetics).

The diagram segments a fish into several key anatomical parts (head, backbone and thorns, skin and fins, swim bladder, viscera, and muscle), listing for each part specific valuable compounds. Specifically, the head (green circle) is indicated as a source of oils (e.g., EPA, DHA), proteins, peptides, and HA, while from the backbone and thorns (blue circle), gelatin, minerals (e.g., Ca, P), and collagen can be extracted. Swim bladders (light blue circle) are a valuable source of collagen and other peptides. Skin and fins (yellow circle) are rich in collagen and gelatin, viscera are a significant source of oils like EPA and DHA, protamine, and enzymes, and the muscle may yield, for instance, EPA and/or DHA oils, proteins, and peptides. The visualization emphasized the concept of a “zero-waste” approach in the fishing industry, where by-products are not discarded but rather processed to recover high-value compounds [174,175,176,177].

Fish, beyond their fillet, represent an abundant and underutilized reservoir of diverse bioactive compounds. The by-products generated during fish processing, including skin, heads, bones, viscera, and fins, are increasingly recognized as rich sources of valuable components such as ω-3 fatty acids (e.g., EPA, DHA), high-quality proteins and peptides, collagen, gelatin, minerals, and enzymes. These compounds possess a wide array of health-promoting properties, offering significant potential for integration into functional foods, nutraceuticals, cosmetics, and cosmeceuticals, thereby enhancing sustainability and economic value within aquaculture and fisheries sectors [174,175,176].

#### 3.3.1. General Composition and Nutritional Value of Fish and Its By-Products

**Figure 7:**
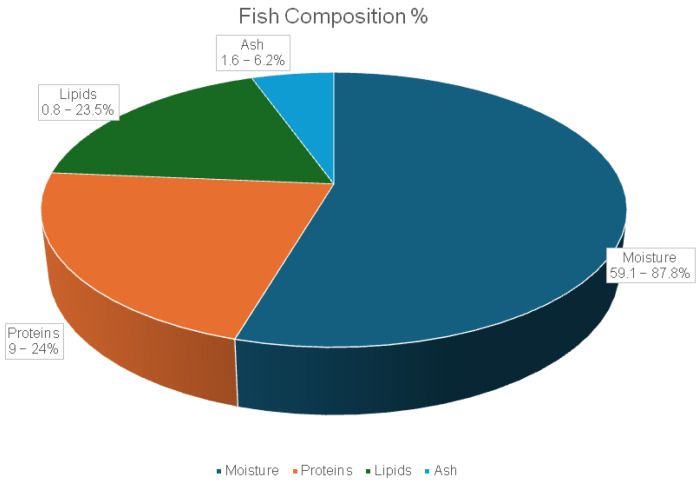
Average (%) chemical composition of fish (Based on data retrieved from https://www.fao.org/4/ae581e/ae581e09.htm#bm9 (accessed on 16 July 2025) [176]).

Fish bioactive compounds are substances derived from fish through biological activity and are recognized for their considerable medicinal and health-promoting potential [178]. The average chemical composition of fish includes 59.1–87.8% moisture, 9–24% protein, 0.8–23.5% lipids, and 1.6–6.2% ash. These values vary depending on the fish species, age, and physiological condition, as estimated by FAO (based on 62 species) (Figure 7) [176,179,180].

Fish also contain essential minerals such as potassium (K), phosphorus (P), sodium (Na), zinc (Zn), magnesium (Mg), calcium (Ca), iron (Fe), selenium (Se), and iodine (I) [181,182]. Fish flesh is also rich in proteins, several amino acids, PUFAs, minerals, micronutrients, and dietary vitamins A, B_3_, B_6_, B_12_, D, and E [182,183,184]. Moreover, fish oil contains sterols, vitamins, minerals, polyphenols, and pigments, along with fatty acids, including PUFAs (e.g., DHA, EPA, arachidonic acids), monounsaturated fatty acids (MUFAs, e.g., gondoic, palmitoleic, and oleic acids), and saturated fatty acids (SFAs, e.g., stearic and palmitic acids) [176,185,186,187]. Fresh, dried, fermented, and salted fish exhibit high lipid content, as well as a wide range of macromolecules, minerals, and amino acids [188,189]. Finally, calcium has been found in fish bones, crude enzymes were extracted from the viscera, and proteins were traced in both fish skin and head [44].

#### 3.3.2. Protein-Based Bioactive Compounds

Fish protein hydrolysates (FPH) and hydroxyapatite (HAP) derive from fish proteins and require controlled enzymatic hydrolysis to become active. They are primarily obtained from muscle, skin, and waste (e.g., head, trimmings, fins, viscera, frames) [175]. Muscle tissue contains ~65–75% of the total fish protein [190].

Bioactive peptides (protein fragments), consisting of 2–20 amino acids, exist in fish proteins in an inactive form [175] and are released through in vivo proteolysis or in vitro enzymatic hydrolysis [175,191]. Fish proteins are therefore rich in essential (e.g., leucine, lysine) and non-essential (e.g., aspartic, glutamic) amino acids. For instance, protein-rich fish by-products such as backbone, skin, head, viscera, and blood can be used to formulate collagen, gelatin, and proteoglycans [178].

#### 3.3.3. Fatty Acid (Lipid) Content

Fatty acids are composed of carbon atoms with a carboxyl group (COOH) at one end and a methyl group (CH_3_) at the other end. Based on saturation (e.g., length, double bonds, and hydrogen atom arrangement), they are classified as SFAs (such as stearic and palmitic acids), MUFAs (such as gondoic, oleic, and palmitoleic acids), and PUFAs (such as EPA, DHA, α-linoleic acid (ALA), and arachidonic acid). PUFAs are essential fatty acids (EFAs, ω-3 and ω-6) not synthesized in the body that must be obtained via diet [176,192,193]. EPA, DHA, and ALA are the major ω-3-fatty acids, with ALA specifically acting as the precursor in the body’s production of EPA and DHA [194]. PUFA content is heavily influenced by temperature, water salinity, bioecology, and diet (e.g., algae, plankton) [192,195].

Fish lipid content is another equally important factor, as they can be categorized into very low fat, low fat, medium fat, and high fat depending on whether we are dealing with wild or farmed fish [195]. Moreover, marine fish oils, particularly from the liver, head, and viscera, are rich in PUFAs (mainly ω-3) and vitamins A, D, and E [44].

#### 3.3.4. Fish Polar Lipids

Numerous studies have demonstrated the critical roles of ω-3 PUFAs at the cellular level: they preserve membrane homeostasis, regulate gene expression, and maintain a healthy balance with ω-6 PUFAs to control inflammatory responses. Most clinical trials have used ω-3 PUFAs in triacylglycerol (TAG) or ethyl ester form. More recently, fish products enriched with ω-3 PUFAs linked to phospholipids (PLs) have shown promising health benefits in clinical findings [196].

**Figure 8:**
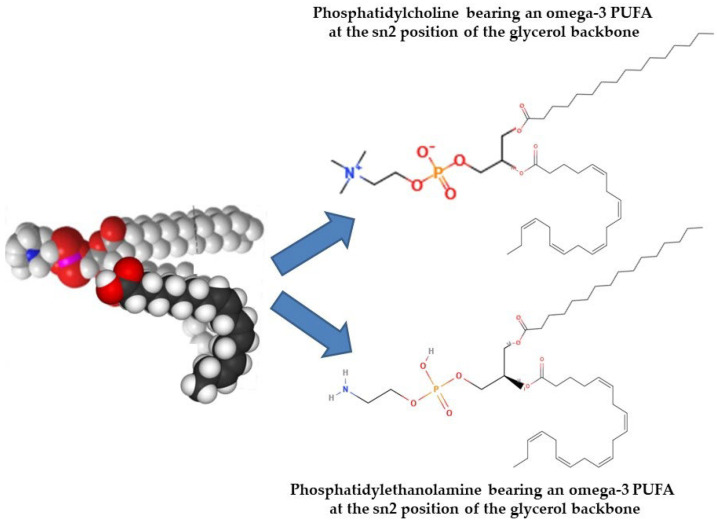
Structures of representative fish PL bioactives with anti-inflammatory and antithrombotic health-promoting properties, according to Tsoupras et al. 2022 [196] (Structures depicted were reproduced from https://molview.org/ and https://smart.servier.com/, both accessed 25 June 2025. (PUFA = polyunsaturated fatty acids)).

Naturally, ω-3 PUFAs occur in two primary forms: free form (from partial hydrolysis) and esterified (bound to PLs or TAG). Typically, PLs contain two fatty acids esterified to a glycerol or sphingosine backbone, which is further esterified to a phosphate and polar head group. In contrast, TAGs consist of three fatty acids bonded to glycerol via ester linkages. TAGs are highly hydrophobic, whereas PLs are amphiphilic (having both polar heads and nonpolar fatty acids), allowing them to form micelles and liposomes and underlie their biological significance. Although bioactive PLs are present in fish and marine foods at lower levels compared to TAGs, the PLs that do exist, particularly glycolipids or phospholipids carrying ω-3 PUFAs, exhibit strong anti-inflammatory and anti-thrombotic effects. Based on their polar head group, glycerol-based phospholipids can be divided into several subgroups (Figure 8) [197,198,199,200].

Oily fish such as salmon, herring, and mackerel are primary sources of marine phospholipids. Their glycerol-based phospholipids make up the majority of the classes of significant fish phospholipids, with trace levels of ether glycerol-based and sphingolipid-based phospholipids. Choline, ethanolamine, serine, glycerol, or inositol can all be found in the head group. Glycerol-based phospholipids, especially phosphatidylcholine (PC), are most abundant, along with lesser amounts of phosphatidylethanolamine (PE), phosphatidylinositol (PI), phosphatidylserine (PS), lysophosphatidylcholine (lyso-PC), and sphingomyelin. PC, which is rich in ω-3 PUFAs at the sn-2 position of the glycerol backbone, is the main PL class of oily fish-derived PLs [198,200,201]. In these PLs, EPA and DHA are the main n-3 PUFAs found, while docosapentaenoic acid (DPA) and stearidonic acid are traced in smaller amounts [202]. Depending on species, up to one-third of EPA and DHA in fish may be found in PL form [196].

Glycolipids, either sphingo-based or glycerol-based, also appear in marine sources [203]. Glycerol-based glycolipids consist of fatty acids attached at positions two and three of the glycerol backbone (nonpolar group), while a mono- or oligosaccharide (polar group) is linked at position one (Figure 8). Sphingo-based versions include a sphingosine backbone, a fatty acid chain, and a polar sugar group. These sphingo-based glycolipids are a more complex subclass of PLs. Many marine organisms own a sulfate group attached to the sugar one; these are also abundant in marine sources, particularly microalgae, and hence pass on to fish through the food chain [204,205].

Marine glycolipids are essential for cellular recognition and cell–cell interaction processes, due to their significant role in providing carrier molecules with certain biological, physical, and chemical characteristics [203]. Therefore, they possess diverse bioactivities such as antibiotic, antimalarial, antiviral, antitumor, immunomodulatory, and neurogenic actions [203]. Plus, fish glycolipids [206], as well as microalgae, including those having a sulpho-group, possess excellent anti-inflammatory and antithrombotic benefits [204,205].

Marine PL digestion and absorption mainly take place in the small intestine, unlike TAGs. PLs bypass lingual and gastric lipases and are efficiently hydrolyzed by pancreatic phospholipase A_2_ (PLA_2_) in the small intestine [207]. PLA_2_ hydrolyzes most PLs in the sn-2 position in the lumen. As a result, lysophospholipids (lyso-PLs) and free fatty acids (FFAs) are absorbed by enterocytes, re-esterified (although some FFAs are incorporated into TAG), and sent into circulation mainly via chylomicron surface layers (as opposed to TAGs, which are incorporated into chylomicron cores) and, to a lesser extent, very low-density lipoproteins (VLDLs). Once degradation of the TAG-rich chylomicron particles takes place, PLs and their intact fatty acids can be taken up by high-density lipoprotein (HDL) rapidly (within five to six hours following PL consumption). HDL may then transfer both PLs and their fatty acids into the cells of many tissues and organs [202].

Remarkably, about 20% of intestinal marine PLs are directly integrated into HDLs without hydrolysis [202]. This contributes to the efficient distribution of PUFAs to critical tissues, including the heart, liver, brain, lungs, erythrocytes, and platelets [202,207]. Therefore, dietary PUFAs bound to PLs affect the composition of both HDL and LDL differently compared to TAG-bound PUFAs, which are typically located in the core of these lipoproteins. Marine PLs rich in PUFAs have distinct effects on lipoprotein composition and activity, their distribution throughout the body, and the incorporation of fatty acids into tissues. The levels and function of HDL, which is responsible for eliminating excess cholesterol from the bloodstream and atherosclerotic plaques, are directly impacted by dietary marine PLs, which are preferentially incorporated into the surface of HDLs. These PLs also possess potent anti-inflammatory and antioxidant properties, helping to reduce LDL oxidation. This, in turn, reduces the platelet-activating factor (PAF) generated from oxidation and plasma oxidized PAF-like lipids, ultimately preserving endothelial cell homeostasis and safeguarding the cardiovascular system [197,207].

When bioactive fish PLs are compared to their neutral forms, such as TAG or lipid esters of ω-3 PUFAs, the former exhibit higher bioavailability of ω-3 PUFAs. This is attributed to their amphiphilic character, enabling them to travel within plasma lipoproteins and be more readily integrated into cell membranes. Examples of bioactive PLs include phospholipids and glycolipids carrying ω-3 PUFAs in their structure [197,199,207]. Fish PLs are rich in ω-3 PUFAs and exhibit anti-inflammatory properties and improved neural function. It is suggested that fish PLs can transport ω-3 PUFAs more efficiently [207,208], enhancing their anti-thrombotic and anti-inflammatory efficacy [198,200,201,206,209,210].

**Figure 9:**
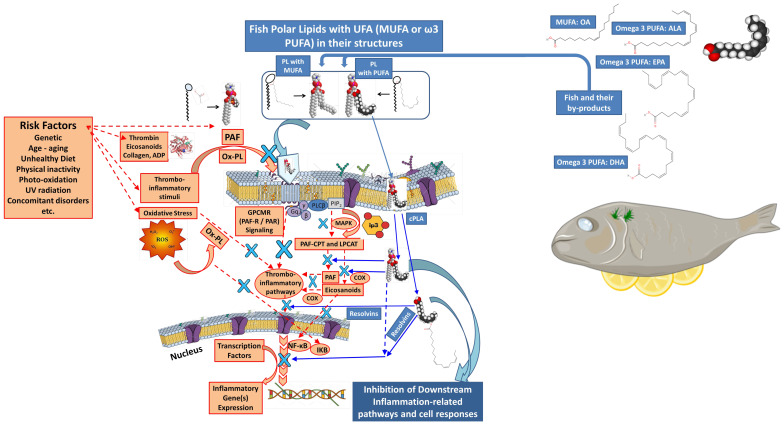
Mechanisms of action of biofunctional polar lipids with UFA (MUFA or ω-3-PUFA) in their structures, derived from fish and their by-products, against thrombo-inflammatory signaling and associated inflammatory cell responses and manifestations. (Red colors: representative signaling of thrombo-inflammatory stimuli induced by the presence of several risk factors, such as those of PAF and thrombin, which, via the pathways shown, propagate the inflammatory cell responses. Blue colors: bioactive polar lipids (PLs) rich in UFA (MUFA or ω3-PUFA) from fish or their by-products can beneficially affect all these signaling pathways and transcription factors and thus further inhibit the expression of thrombo-inflammatory genes and associated cell responses (the blue X represents an inhibitory effect on a pathway and/or an enzyme and/or a receptor and/or a transcription factor and/or the expression of thrombo-inflammatory genes by fish-derived bioactive polar lipids, as indicated by the blue arrows). Abbreviations: UFA = unsaturated fatty acid; MUFA = monounsaturated fatty acids; PUFA = polyunsaturated fatty acids; PAF = platelet-activating factor; GPCMR = G-protein-coupled membrane receptors; PAF-R = PAF-receptor; PAR = protease-activated receptors for thrombin; PAF-CPT and LPCAT = the basic biosynthetic enzymes of the two distinct pathways of PAF-synthesis, PAF-cholinephosphotransferase and lyso-phosphatidylcholine acetyltransferase 2, respectively; cPLA = cytoplasmic phospholipase A2; MAPK = mitogen-activated protein kinase; IP3 = inositol trisphosphate; OA = oleic acid; ALA = alpha-linolenic acid; COX = cyclooxygenase; NF-kB = nuclear factor kappa beta; IKB = inhibitor of NF-kB; ROS = reactive oxygen species; PL = polar lipids; Ox-PL = oxidized phospholipids; ADP = adenosine diphosphate; PLCβ = phospholipase C beta (Cell/nucleus membranes/structures depicted were reproduced from https://mindthegraph.com/ and https://smart.servier.com/, both accessed 25 June 2025. Information on the mechanisms involved and the activities of fish PLs were retrieved from Tsoupras et al., 2022 [196]).

Moreover, marine PLs are less susceptible to oxidation compared to ω-3 PUFAs [207,211]. This may be due to the presence of naturally occurring polar antioxidants within the PL structure in cells and food. Examples include astaxanthin, found in salmon and microalgae, and lipid-soluble vitamins A, D, and E (Figure 9).

**Figure 10:**
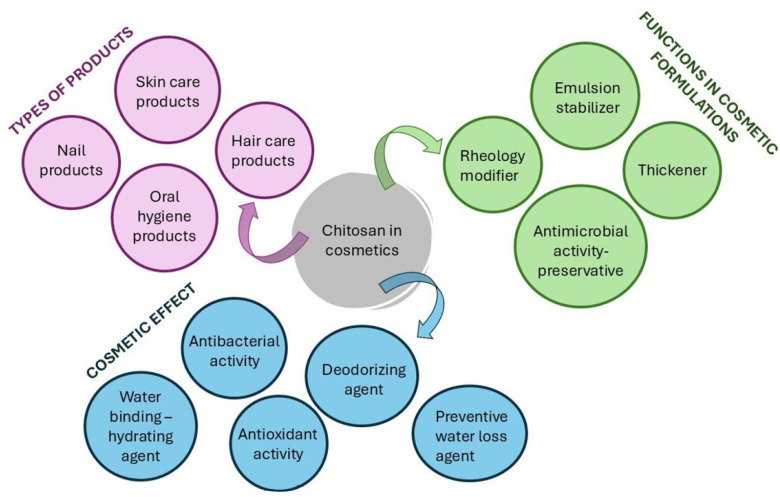
A summary of chitosan applications in cosmetics.

PLs themselves also provide excellent health benefits independent of their ω-3 PUFA content. Specifically, they have been shown to improve cognitive function [139,212,213] and reduce arteriosclerotic plaque formation [214]. The latter occurs by lowering PAF level, an inflammatory and thrombotic mediator, and consequently reducing its atherogenic effect [206]. Additionally, fish PLs inhibit both the PAF and thrombin pathways and reduce platelet agonists like collagen and adenosine diphosphate (ADP), which are involved in platelet activation and aggregation [198,200,201]. Marine polar lipids are more effective than triglycerides in transporting PUFAs to various organs [208], owing to their higher anti-inflammatory and anti-thrombotic efficacy (Figure 9) [197]. Marine fish species such as sea bass (*Dicentrarchus labrax*) and sea bream (*Sparus aurata*) contain bioactive PLs that are highly effective against the PAF mediator [215]. They can also inhibit the enzymatic activities of regulatory enzymes involved in PAF biosynthesis (Figure 10) [210] and exhibit excellent anti-atherogenic and antitumor effects [214].

Oily fish, such as salmon and herring, are rich in ω-3 PUFAs and display strong anti-inflammatory and anti-thrombotic properties. They also have a low ω-6/ω-3 PUFA ratio, which contributes to the superior anti-inflammatory and cardioprotective function of fish PLs. A lower ω-6/ω-3 PUFA ratio is associated with greater inflammatory protection [198,200,201,209]. Transport of ω-3 PUFA-rich PLs occurs via plasma lipoproteins to cell membranes. There, PLA_2_ catalyzes the release of ω-3 PUFAs from the sn-2 position of PLs. The released ω-3 PUFAs subsequently act through eicosanoid pathways (COX enzymes) to resolve inflammation and modulate the inflammatory cell response (Figure 10).

Mackerel is another oily fish that contains similar bioactive PLs. These bioactive PLs exhibit potent anti-thrombotic and anti-inflammatory properties against both PAF and thrombin pathways. Studies have shown that they are stronger anti-PAF and anti-thrombin agents compared to neutral lipids, making them more anti-atherogenic [216].

Sardines, also an oily fish, are rich in ω-3 PUFAs and show strong activity against PAF. Raw sardines possess a high ω-3 PUFA content and a very low ω-6/ω-3 PUFA ratio, factors that explain their notable anti-inflammatory and cardioprotective properties [217]. Beyond oily fish, cod fish, a lean white fish, also contributes valuable biofunctional lipids. Cod PLs have demonstrated the most promising anti-platelet activity against aggregation induced by PAF and thrombin. Thus, raw cod fish exhibits significant anti-atherogenic and cardioprotective properties [218].

#### 3.3.5. Vitamin and Mineral Content

Fish provide significant amounts of vitamins A, D_3_, E, and B-complex vitamins, mainly via fish oil consumption [176,193,219]. Furthermore, marine species have particularly high levels of essential and trace mineral elements. Compared to terrestrial meals, seafood contains several vital minerals in higher quantities, especially in fish flesh (e.g., sodium (Na), potassium (K), calcium (Ca), magnesium (Mg), phosphorus (P), sulfur (S), iron (Fe), manganese (Mn), zinc (Zn), copper (Cu), selenium (Se), fluorine (F), and iodine (I)) [193,220,221], while fish bones have displayed high Ca, P, and hyaluronic acid (HA) content [44].

#### 3.3.6. Pigments and Carotenoids

Pigments are responsible for the wide range of colors in fish, making them important quality assessment factors. Common fish carotenoids include lutein (green-yellow, in mainly freshwater species), astaxanthin (yellow, red-pink in salmon, in both marine and freshwater species), canthaxanthin (orange-red), zeaxanthin (yellow-orange), β-carotene (orange), α/β-doradexanthins (yellow), and tunaxanthin (common in yellow fish species, like scombriana, caragana, percina, and *Seriola quinqueradiata*) [192]. Certain carotenoids are unique to particular fish species, and their typically smaller concentrations vary with the fish’s nutrition and physiological circumstances [222].

#### 3.3.7. Collagen and Gelatin

Fish by-products, including skin, bones, and cartilage, are abundant sources of collagen [18,47,223]. It is estimated that these filleting residues can constitute up to 75% of the fish’s total weight, with skin and bones making up a significant portion of this collagen-rich mass [224]. Collagen is a primary structural and fibrous protein that forms the extracellular matrix in fish, supporting the physiological function of tissues in the head, cartilage, skin, tendons, and bones. Composing 25% of the total protein in fish, it is the most abundant single protein. While various collagen types exist, Type I is the most common in fish by-products, found specifically in connective tissue, muscles, skin, bone, and corneas [225,226,227,228]. Furthermore, collagen boasts a high content of hydroxyproline, proline, and glycine, which are denatured when dilute acids are present and converted into soluble proteins such as gelatin [224].

When collagen is subjected to acid or alkali pretreatment followed by heating above the transition temperature, its triple helix polypeptide chains denature, resulting in the formation of gelatin [175,192]. Gelatin is a colorless, tasteless, proteinaceous macromolecule or a biopolymer. Due to its structural similarities with collagen, gelatin shares many of its properties, making it suitable for creating edible and biodegradable films that can extend the shelf life of food products [178,192,224].

The characteristics of gelatin, however, depend on the type of fish it originates from. For example, gelatin derived from cold-water fish typically exhibits lower industrial value compared to that from moderate- or warm-water fish. This difference stems from the decreased heat stability of collagen in cold-water species, which is influenced by the lower content of proline and hydroxyproline in their protein macromolecules [229]. Interestingly, gelatin from warm-water fish shows many similarities with porcine gelatin, suggesting its potential as an alternative to mammalian gelatin in pharmaceutical products. Conversely, gelatin from cold-water fish can be effectively used in microencapsulation of vitamins and other pharmaceutical applications [192].

While fish skin was traditionally the primary source, current research indicates that various other fish by-products are rich in gelatin. Notable examples include gelatin extracted from the skins of Pacific cod (*Gadus macrocephalus*) and seabass (*Lates calcarifer*), as well as from the scales of bighead carp (*Hypophthalmichthys nobilis*), the heads of mackerel (*Scomber scombrus*), and the bones of black tilapia [37,230,231,232].

#### 3.3.8. Glycosaminoglycans: Chondroitin, Glucosamine, and Hyaluronic Acid

The extracellular matrix (ECM) and the synovial fluid are primarily composed of glycosaminoglycans (GAGs), including chondroitin sulfate (CS), glucosamine sulfate (GS), and hyaluronic acid (HA). GAGs are anionic heteropolysaccharides characterized by repeating disaccharide units of amino sugars and uronic acid, linked by glycosidic bonds. Their fundamental roles include cellular signaling, tissue hydration, and maintaining structural integrity [233]. Chondroitin sulfate, a key GAG, can be effectively extracted from marine sources, notably from blue shark (*Prionace glauca*) cartilage, which is a rich and sustainable reservoir of CS. Marine-derived CS often exhibits a lower molecular weight compared to its terrestrial counterparts, enhancing its bioavailability and absorption. Studies have demonstrated that CS from blue shark cartilage, due to its amino acid constituents, possesses anti-inflammatory and chondroprotective activities, contributing to the maintenance of joint health and cartilage regeneration, while also finding applications in cosmeceuticals for skin hydration and anti-aging formulations [234,235,236].

GAGs are broadly categorized into sulfated and non-sulfated types. For instance, CS, dermatan sulfate (DS), keratan sulfate (KS), and heparin/heparan sulfate (HS) are sulfated GAGs, whereas HA is a non-sulfated type [237]. CS is abundantly found in the ECM of connective tissues such as the brain, skin, tendons, and cartilage [238]. Notably, CS can be specifically extracted from various fish parts, including the head, skin, eyes, cartilage, and fins [239,240]. Moreover, glucosamine is a crystalline substance widely distributed throughout connective tissue and cartilage, particularly as a component of chitin. It serves as an important precursor for the biosynthesis of GAGs [192,241]. Finally, HA, as a main ECM component, is found in high concentrations in the synovial fluid, skin, and vitreous body [240,242]. Valuable compounds derived from fish processing by-products are discussed in Table 3.

## 4. Cosmetic Applications of Marine By-Products

Marine by-products such as fish skin, bones, scales, crustacean shells, and mollusk tissues are increasingly utilized in cosmetics due to their richness in bioactives such as collagen, chitin, peptides, minerals, and ω-3 fatty acids. These compounds offer anti-aging, moisturizing, antioxidant, UV-protective, and skin-repairing properties, making them valuable ingredients in creams, serums, masks, and sunscreens. Their incorporation not only enhances product functionality but also supports sustainability by valorizing seafood waste [11,28,78,79]. Table 4 provides examples of commercial cosmetics and cosmeceuticals that utilize marine by-products as cosmetic ingredients.

### 4.1. Marine Polysaccharides as Cosmetic Ingredients

#### 4.1.1. Marine Chitin and Its Derivatives as Cosmetic Ingredients

Polysaccharides play a vital role in cosmetics, serving as moisturizers, emulsifiers, thickening agents, and wound-healing agents due to their broad spectrum of bioactivities. These include antimicrobial, antioxidant, anti-aging, metalloproteinase (MMP)-inhibitory, anticancer, anti-inflammatory, antifungal, and other therapeutic properties. Among them, chitin, chitosan, and their derivatives have garnered special attention for cosmetic use [117,265,266]. These compounds have been proposed for use in formulations targeting the skin, oral cavity, nails, and hair, allowing the treatment of various related conditions [117].

Chitin is a rigid polysaccharide with low solubility, making it challenging to work with. In contrast, chitosan is soluble in acidic solutions and can form films, scaffolds, fibers, and micro-, nano-, and milliparticles. At physiological pH, certain oligosaccharides and derivatives of chitin and chitosan are water-soluble, exhibiting enhanced or novel properties. In cosmetics, chitin, chitosan, and their derivatives act either as active ingredients due to their bioactivities or as carriers for other bioactive agents owing to their technological adaptability [35].

In the European Union (EU), cosmetic ingredients are regulated under the Cosmetic Product Regulation (2009) (https://health.ec.europa.eu/system/files/2016-11/cosmetic_1223_2009_regulation_en_0.pdf, accessed on 6 June 2025)). The CosIng database lists eight entries under “chitin”, mostly cited as abrasive and bulking agents. The search term “chitosan” yields 55 entries, where chitosan is primarily characterized as a film-forming and hair-fixing agent [267].

Chitosan and its derivatives are excellent candidates for skincare formulations due to their high molecular weight (HMW) and cationic nature, which promote skin adhesion [78]. Their safety, nontoxicity, biocompatibility, and biodegradability make them suitable as excipients or active ingredients in cosmetics [117]. They are used in various cosmetic products, including lotions, skin moisturizers, creams, foundations, eye shadows, lipsticks, cleansing wipes, and bath products [268]. A summary of well-documented chitosan applications in cosmetics is depicted in Figure 10.

##### Chitosan as an Anti-Aging and Moisturizing Agent

Skin aging may be intrinsic or extrinsic, with UV radiation, smoking, and pollution being major extrinsic factors. The primary signs of skin aging include dryness, relaxation, roughness, and laxity of skin tissue, which, along with UV radiation exposure, are among the most well-established causes of wrinkles and hyperpigmentation, resulting in photo-aging. Chitosan, especially HMW types, forms a transparent film on the skin, reducing cutaneous water loss and improving skin elasticity and smoothness; therefore, it is a prominent cosmetic moisturizing agent (Figure 10) [117].

Morganti et al. [269] demonstrated that chitin and chitosan nanofibril-hyaluronan carriers, either as nanoemulsions or non-woven films, increased skin collagen formation and inhibited collagenase activity. Results indicated that lutein was completely released from chitosan-hyaluronan nanocarriers within 20 h, while the chitin-hyaluronan nanocarriers required almost twice that duration. In vitro tests revealed that nanoemulsions containing an active liposomal complex (hyaluronic acid-phosphatidylcholine-chitin) elevated skin collagen generation. Conversely, a nanoemulsion enriched with the antioxidant complex melatonin-vitamin E-β-glucan (MEB) exhibited potent collagenase inhibition (~90%). Further in vitro assays showed that oral and topical application of chitin-nanofibril-hyaluronan MEB promoted skin repair in areas affected by acne and photo-irradiation, while also rebalancing dermal moisture. Morganti et al. also investigated the efficacy and safety of anti-aging beauty masks composed of tissues containing chitin nanofibrils (CN) and nano-lignin (LG), derived from crustaceans and plant biomass. Their findings revealed that these masks repressed matrix metalloproteinase-1 (MMP-1) and increased collagen type I, thereby reducing skin aging. Early in vivo outcomes validated the preventative and rejuvenating properties observed in an in vitro study involving 30 women with signs of photo-aging [270].

Afonso et al. [271] revealed that re-acetylated chitosan films released antioxidants with potential anti-aging value, such as vitamin C, more effectively, offering flexibility, non-cytotoxicity, and faster release (~52% of the active ingredients, in 15 min). Therefore, chitosan films could be useful as anti-aging agents in skin masks. Chaiwong et al. [272] reported that carboxymethyl chitosan (CMCH) with different molecular weights significantly improved water solubility and skin hydration. More specifically, low MW-CMCH, moderate MW-CMCH, and high MW-CMCH improved water solubility by 96, 90, and 89%, respectively, compared to chitosan. In vitro testing on pig skin using a Corneometer^®^ showed that 0.5% high MW-CMCH had a higher moisturizing effect. High MW-CMCH is a high-viscosity, water-soluble polymer with potential for use as a thickening agent and emulsion stabilizer in cosmetics. Additionally, Chen et al. [273] created montmorillonite-quaternized CMCH composites that promote UV protection and moisture retention, serving as an effective anti-aging cosmetic ingredient.

##### Chitosan as a UV-Radiation-Protective Agent

Chitin and chitosan form stable, water-resistant, cytocompatible films that absorb UV below 400 nm, which are a perfect candidate for protective cream formulations [117]. UV light, more specifically UV_A_ (320–400 nm) and UV_B_ (290–320 nm), represents the primary components of solar radiation. These types of radiation can induce various negative skin reactions, including sunburn, skin degeneration, photosensitivity, phototoxicity, photo-aging, immunosuppression, and skin cancer [182,274]. At this point, it must be addressed that squid pens can be a valuable source of chitosan, which can be further utilized for the development of scaffolds or anti-UV biomedical products [275]. The well-known adhesive properties of chitin and chitosan are evident from their interaction with positively charged polysaccharides and negatively charged keratin-based structures [276]. Therefore, to prevent negative skin reactions caused by prolonged sun exposure, sunscreens incorporating chitosan and other highly protective ingredients are utilized [277,278].

According to Ntohogian et al. [278], chitosan nanoparticles have been utilized as active carriers and stabilizers for encapsulating natural and purified annatto and saffron (sunscreen agents) to formulate UV-protective sunscreen emulsions. Their study demonstrated that the prepared emulsion showed low cytotoxicity and good stability during 90 days of storage. The formulas displayed sun protection factor (SPF) values ranging from 2.15 to 4.85.

Morsy et al. [279] investigated the development of a multifunctional hydroxyapatite-chitosan (HAP-chitosan) gel designed to function as an antibacterial sunscreen. In this formulation, HAP nanoparticles were uniformly distributed throughout the chitosan matrix. The application of a film made from this hybrid system would thus reduce the negative effects after exposure to UV radiation.

##### Chitosan as a Skin-Cleansing Agent

Skin-cleansing cosmetic formulations aim to rid the skin of foreign substances resulting from environmental exposure or cosmetic product application. Given that chitosan and its derivatives are cationic, they can be utilized as positively charged vehicles for delivering personal cleaning products. Consequently, leveraging the interaction between the positive charges of the chitosan backbone and the anionic charges of the skin’s surface presents a highly promising method for ensuring the targeted release of cleaners [280,281].

According to Tangkijngamvong et al. [282], chitosan-containing cleansers in the form of nanoparticles can be used as sebum-controlling formulations, as both chitosan particles (CP) and CP-proretinal nanoparticles (PRN) have shown a decrease in sebum levels. Furthermore, CP-PRN also showed improvement in skin redness. Theerawattanawit et al. [283] found that chitosan gels could be effectively utilized in reducing sebum levels without adverse side effects. Chitosan is capable of forming complexes with sebum, thereby facilitating its removal. Concurrently, chitosan can establish a barrier that inhibits sebum formation on the skin’s surface [281].

##### Chitosan as an Antibacterial Agent

Positively charged chitosan can interact with negatively charged bacterial cell walls, initiating a weakening and shrinking process. This is a widely accepted mechanism for explaining the topical antibacterial activity of chitosan. The inactivation of the bacterial cell is dependent on the molecular weight and charge density of the chitosan chains. Notably, the antibacterial activity of chitosan increases with its molecular weight [284]. Verma et al. [285] proved the antibacterial role of chitosan by observing over a 90% decrease in bacterial proliferation after chitosan treatment.

Deng et al. [286] studied the application of a multifunctional, bio-based hydrogel composed of quaternized chitosan and dialdehyde bacterial cellulose. This hydrogel exhibited good wound-healing and antibacterial activity. In another study, Liu et al. [287] formulated a hydrogel from borax cross-linked polyvinyl alcohol (PVA) and CMCH. This composition demonstrated excellent self-healing properties. Furthermore, the addition of highly stable silver nanoparticles (AgNPs) to the hydrogel was proved very effective in inhibiting bacteria like *Escherichia coli* (*E. coli*) and *Staphylococcus aureus* (*S. aureus*), making it applicable for both wound-healing and antibacterial properties.

##### Chitosan as a Nail-Care Agent

The nails are an extension of the skin, functioning as a structure produced by the skin itself [288]. Onychomycosis, a fungal infection of the nail unit, is classified distinctly from other skin conditions. Treatment typically involves the topical application of broad-spectrum antimycotics, sometimes combined with systemic oral medication. The most common causes of onychomycosis are skin infections and nail trauma, both of which alter the nail’s natural barrier. Nail trauma can result from either mechanical injury or exposure to chemical agents [289]. A nail lacquer, a topical agent, is a successful formulation for preventing fungal infections compared to creams and solutions, primarily due to its stability to remain at the site of action for a longer duration [290].

Hydroxypropyl chitosan (HPCH), a semisynthetic derivative of chitosan, has revealed effectiveness in delivering active ingredients to nails. It acts as a protective layer, maintaining nail structure, safeguarding keratin, and keeping nails hydrated, while also reducing symptoms of dystrophy in psoriatic nails [291,292]. Compared to typical cosmetic treatment chemicals like isopropyl alcohol or urea, repeated application of HPCH nail solution can prevent the occurrence of new or recurrent fungal infections. Moreover, it can enhance the hardness, tensile strength, and flexural strength of hoof samples compared to untreated controls. Additionally, HPCH decreased the sample’s crumb area following dermatophyte hyphae penetration and abrasion [293].

##### Chitosan as a Hair-Care Agent

The piliferous ends of hair, which grow at the skin’s surface, are primarily composed of solid proteins, notably keratin. Keratin is rich in amino acids such as cysteine and lysine, and hair also contains melanin, which imparts its color [117]. Hair damage can arise from high temperatures during drying, contact with aggressive chemical agents during coloring, and exposure to UV radiation or chlorine, leading to both chemical and environmental damage [294]. The use of chitin and chitosan offers unique properties beneficial for hair, including improved hydration, assistance in rebuilding damaged hair, and enhanced healthy shine. Chitosan is extensively incorporated into a wide variety of hair care products, including shampoos, rinses, permanent wave agents, hair sprays, hair colorants, hair tonics, and styling lotions, owing to its capacity to enhance the rheological characteristics of cosmetic formulations or improve the adherence of specific ingredients to the hair [35,281,295,296,297,298].

Conditioning is a prevalent hair care procedure that utilizes cationic polymers, such as chitosan. The proteinic structure of damaged hair is characterized by a denatured state with negative charges unable to be biologically repaired. Consequently, physicochemical techniques are necessary for long-term hair repair, leveraging cationic polymers’ ability to form a film on negatively charged surfaces [299,300]. Chitosan and its cationic derivatives interact with the negatively charged keratin surface of damaged hair, forming a transparent, elastic film that results in softer and stronger hair [301].

Mohamed et al. [298] studied the creation of organic hair conditioners (OHC) based on two chitosan–thiadiazole conjugates, chitosan–(ethylthio-thiadiazole) (CH-ETD) and chitosan–(benzylthio-thiadiazole) (CH-BTD), combined with natural fragrances. Their study revealed that the pH of all OHC ranged between 4.2 and 4.7, an acceptable range to avoid skin irritation. Moreover, three specific formulas (CH, E2, and B2) demonstrated optimal efficacy in retaining moisture, detangling hair, facilitating styling, preventing inflammation or irritation, minimizing fizz, and creating a protective barrier on the hair.

Sionkowska et al. [296] explored the effects of ternary blends, including chitosan, collagen, and hyaluronic acid, for their promising hair-conditioning properties. They found that chitosan can enhance the mechanical properties, surface free energy, and stability in aqueous conditions of conditioning deposits, hence improving the appearance of hair fibers. When combined in a conditioning formulation, chitosan, HA, and collagen effectively increase elasticity and resistance to hair damage.

In addition to chitosan and its derivatives, other peptide-based technologies such as Olaplex^®^ and K18^®^ are also employed in hair care to enhance hair health and repair. These systems utilize bioactive peptides that penetrate the hair cortex to repair disulfide bonds and polypeptide chains, complementing the surface-level film-forming and conditioning effects provided by chitosan-based systems. Thus, while chitosan offers structural reinforcement and conditioning on the hair surface, peptide-based treatments contribute to molecular-level repair within the hair shaft, creating a comprehensive approach to their health management [302].

##### Chitosan as an Oral-Cavity-Protective Agent

The organs within the mouth, including teeth and gums (soft connective tissues surrounding the teeth and covering the alveolar process), are central to dental care [117]. Various dental diseases, such as dental caries, anodontia, tooth wear, bruxism, periodontitis, and gingivitis, can affect both teeth and gums. To prevent conditions like oral mucositis, plaque formation, bacterial growth, and periodontal issues, chitosan and its derivatives are incorporated into gels, sprays, chewing gums, mouthwashes, dentifrices, and microspheres [303,304,305].

In oral healthcare, polymers, particularly chitosan, can function either as a vehicle or as a carrier. Examples of the former include their use in toothpaste, mouthrinse, chewing gum, and dental vanishes, while they can also act as a carrier for sodium fluoride (NaF), herbal extracts, and chlorhexidine. Chitosan’s activities in the oral cavity include buffering mouth pH, antimicrobial action, inhibiting biofilm adsorption, and acting as an anti-abrasive agent [35].

Ganguly et al. [306] described the preparation of periodontal gels by using natural polymers like badam gum, karaya gum, and chitosan. Their study concluded that the surface pH values of these gels varied based on the type and concentration of polymer used. Furthermore, the gels had excellent antimicrobial activity upon the addition of moxifloxacin hydrochloride, suggesting their potential as vehicles in treating periodontitis.

Nguyen et al. [307] developed fluoride-loaded nanoparticles based on chitosan, alginate, and pectin polymers for dental delivery. Using NaF as an active ingredient, chitosan enabled the formation of stable, spherical, and monodisperse nanoparticles. The advantage of this formulation lies in its ability to provide a low-concentration but continuous delivery of fluoride from the chitosan nanoparticles, with potential for accelerated release in an acidic environment, making it promising for dental caries prevention. Samiraninezhad et al. [308], additionally, created hydrogels from natural polymers like chitosan, alginate, gelatin, and HA, which showed significant promise for delivering oral medications compared to conventional methods. These hydrogels offer sustained drug release, minimized systemic complications, and increased therapeutic efficacy. Consequently, they can be utilized for treating oral lesions.

#### 4.1.2. Marine Hyaluronic Acid, Glycosaminoglycans (GAGs), Carrageenan, and Fucoidans as Cosmetic Ingredients

Marine polysaccharides, except chitin, chitosan, and their derivatives, like marine-derived hyaluronic acid, GAGs, carrageenans, alginates, and fucoidans, have emerged as promising cosmetic ingredients due to their diverse structural features and multifaceted bioactivities. Marine-sourced hyaluronic acid, obtained, e.g., from fish eyeballs, acts as a powerful humectant and dermal filler [309]. Hyaluronic acid retains moisture, supports tissue repair, and reduces the appearance of fine lines and wrinkles owing to its gel-forming, water-binding, skin-rejuvenant, wound-healing, and anti-aging capabilities. Specifically, it forms a film in the stratum corneum that protects the skin but also prevents transepidermal water loss (TEWL), moisturizing the epidermis [2]. For instance, hyaluronic acid from bluefin tuna by-product exhibited notable moisturizing and anti-inflammatory properties, serving as a promising cosmetic and cosmeceutical agent [242].

Hyaluronic acid (hyaluronan) is a non-sulfated glycosaminoglycan (GAG), whereas chondroitin (CS), dermatan (DS), heparan (HS), and keratan sulfates (KS) are sulfated GAGs, all being expressed in the skin. Marine GAGs applied for skin hydration and tissue architecture and can be combined with other polysaccharides for enhanced effects. GAGs, as ECM components, modulate the attraction of skin and bone precursor cells, as well as their differentiation and gene expression. Therefore, they GAGs successfully regulate protein action essential for bone and skin regeneration [310,311,312]. For instance, shark cartilage extracts rich in collagen, glucosamine, and CS have already been applied in several cosmetics [240,313,314,315]. Clinical-grade fish cartilage hydrolysate, rich in CS and collagen peptides, has been shown in randomized, ex vivo supported trials to enhance fibroblast activity, stimulate endogenous hyaluronan and elastin synthesis, and suppress MMPs, ultimately improving skin hydration, elasticity, and barrier function and reducing photo-aging signs [316].

Sulfates, fucose-rich polysaccharides (fucoidan-like molecules), also exist in fish mucus, crustacean exoskeletons, and mollusk tissues. These show antioxidant, anti-inflammatory, and collagen-supporting effects similar to algal fucoidan. In cosmetic formulations, they have been used to reduce UV-induced aging, enhance moisture retention, and stimulate skin repair, making them excellent candidates for serums and anti-aging creams. Animal-origin fucoidans are being explored in pharmaceutical and topical applications, due to their anti-inflammatory, immunomodulatory, and skin-health effects, though most cosmetic research still focuses on brown algae sources [317,318,319]. Moreover, carrageenan derived from red algae has been widely utilized in various applications; however, cosmetic industries still use red algae-based carrageenan, and research is lacking on animal (e.g., fish, crustaceans, mollusks)-source evidence [2,312].

### 4.2. Marine Fatty Acids (ω-3 PUFAs) as Cosmetic Ingredients

The most recent skin-related applications of fatty acids derived from fish oil include their use in the treatment of photo-aging, melanogenesis, dermatitis, cancer, and wounds. The administration of PUFAs has demonstrated efficacy in alleviating symptoms associated with these dermatological conditions. Certain fatty acids are licensed for preventive or therapeutic use in clinical trials. Additionally, some formulations containing fish oil have been approved to treat a variety of skin conditions in both animal- and cell-based research. The main fatty acids obtained from fish oil are linoleic acid (LA), ALA, DHA, and EPA, which will be discussed in the subsequent sections in more detail [320].

Fatty acids significantly influence the maintenance of skin functions. Firstly, they play a crucial role in the regular functions of the skin barrier. Their regulatory role in many different skin conditions depends on their chain length. Long-chain fatty acids help retain hydration, prevent dangerous substances from entering the body, and maintain active metabolic functions in some skin cells. They may also possess anti-inflammatory and hydrating properties. Similarly, medium-chain fatty acids protect against tumor agents and inflammation. Short-chain fatty acids can also trigger an immune response and exhibit anti-inflammatory properties [321].

#### 4.2.1. Fatty Acids as Anti-Photo-Aging Cosmetic Agents

There are two existing cutaneous aging types, chronological and photo-aging. Photo-aging occurs when human skin is continuously exposed to UV sunlight. UV radiation is responsible for causing both acute and chronic adverse skin effects, including photosensitivity, inflammation, immunosuppression, sunburn, and photo-carcinogenesis [320]. Following UV exposure, the skin produces reactive oxygen species (ROS), leading to the infiltration of a large number of neutrophils and macrophages into the skin. Cyclooxygenase-2 (COX-2) is the key protein that mediates inflammatory signals in injuries caused by UV exposure. COX-2 catalyzes the biosynthesis of prostaglandins [322]. Beyond sunscreens, there is a high demand for new agents to provide photoprotection against UV light. Studies have shown that fatty acids are among these agents, proving their ability to photoprotect the skin. More specifically, ω-3 PUFAs can reduce the production of pro-inflammatory eicosanoids by directly competing with the metabolism of arachidonic acid (AA) [320,323]. Additionally, ω-3 PUFAs can inhibit UV-induced keratinocyte responses through the control of COX-2, nuclear factor κΒ (NF-κΒ), and mitogen-activated protein kinase (MAPK)/extracellular signal-regulated kinase (ERK) pathways [324]. Through these processes, fish oil’s ω-3 PUFAs demonstrate promising effects in preventing skin damage induced by UV radiation [325].

Collagen supports the skin and is responsible for skin firmness and elasticity. Type I collagen is the main collagen found in the skin. Normally, collagen is produced by cells called fibroblasts, which are found in high amounts in the dermis. Fibroblasts are also responsible for producing elastin and GAGs. Fibroblasts can be activated and proliferate due to biochemical stimuli, signaling pathways, and the physical tension of the ECM in which they are embedded. Small MW ligands bind to receptors on the fibroblast EC membrane, causing their activation. When fibroblasts are activated, there is an increased production of collagen, elastin, and associated GAGs. Numerous anti-aging techniques aim to influence how fibroblasts produce ECM components. Various ligands, including bioactive peptides, antioxidants, retinoids, vitamins, growth factors, hydroxy acids, a wide variety of plant extracts, and especially ω-6 and ω-3 fatty acids, can affect fibroblast activation and proliferation. Most of these chemicals share the common ability to affect the synthesis of collagen and ECM components either directly or indirectly [326].

Dietary supplementation of certain fatty acids, like arachidonic and palmitic acid, augments skin aging, while topical application of α-lipoic acid (SFA) and cholesterol-phytosterols benefits aged skin. Moreover, topical application of lipids such as ω-3 PUFA is also believed to improve aged skin, implying its efficacy towards enriching fatty acids or sterols with therapeutic benefits and limiting harmful ones [327]. In a cross-sectional study, severe photo-aging was inversely related to higher vegetable oil-, fruit-, and vegetable-derived ALA intake in men. Considering women, photo-aging incidence was inversely associated with higher EPA intake and vegetable oil-derived ALA. Therefore, ω-3 PUFAs supplementation revealed a positive impact on skin aging, yet further epidemiological studies are needed [328].

An anti-aging gel containing EPA and cannabidiol (CBD) reduced the secretion of pro-inflammatory, photo-aging agents prostaglandin E2 (PGE2) and interleukin 8 (IL-8). The application of this cream also resulted in ECM remodeling, reduced crow’s feet wrinkle area and volume, lowered fine-line wrinkle volume, and decreased subepidermal low-echogenic band by 8.8%. It also reduced the red spot area and count and increased skin hydration and elasticity by 31.2% and 25.6%, respectively, after 56 days. Collectively, the ω-3 PUFA-enriched gel enhanced the anti-aging effects and strengthened the beneficial derm-cosmetic properties of CBD-based cosmetics [329].

#### 4.2.2. Fatty Acids as Anti-Hyperpigmentation Cosmetic Agents

Skin hyperpigmentation is a disorder characterized by the formation of dark patches on the skin, which is caused by the excess production of melanin in specific areas. Melanogenesis is the biological process responsible for melanin production [330]. Hyperpigmentation due to melanogenesis can be stimulated by various factors, including UV exposure, growth factors, cytokines, endothelin-1, and α-melanocyte-stimulating hormone (α-MSH) [331]. While DHA does not affect cell viability, it has been shown to promote tyrosinase degradation without altering the expression of the Microphthalmia-associated transcription factor (MITF) and decrease α-MSH-activated melanin synthesis [332]. Studies indicate that ALA and LA can whiten skin by inhibiting tyrosinase [320,333].

Important study findings indicated that 4-*n*-butylresorcinol could effectively regulate tyrosinase activity without any reported toxicity at high concentrations, acting as a beneficial, less-irritative depigmenting agent [334]. Additionally, topical application of linoleic (LA) and α-linoleic (ALA) fatty acids significantly increased the skin lightness value (L*) (from 40.6 of the UV_B_-treated control to 47.1 (ALA) and 48.8 (LA)) and decreased the melanin content by 16.4% and 28.0% after ALA and LA treatment, respectively [320].

#### 4.2.3. Fatty Acids as Anti-Skin-Cancer Cosmetic Agents

Skin malignancies are classified into two main types: melanoma and non-melanoma skin carcinoma (NMSC). UV_B_ radiation is the most common risk factor for skin cancer development [335]. However, UV_A_ has also been shown to contribute to pro-carcinogenic skin development [336]. Oxidative stress and persistent inflammation are the primary pathogenic mechanisms in UV-induced skin photo-carcinogenesis. Additionally, UV exposure significantly induces skin malignancy through the reduction of cutaneous immunity [337]. It has been discovered that the PUFAs in fish oil can prevent cutaneous carcinogenesis during both the initiation and promotion phases.

Fatty acids exert various beneficial effects on melanoma, as palmitic acid increases melanin levels, and DHA may suppress tumor proliferation. SFAs and PUFAs demonstrate opposing effects on melanin content in melanoma cells, while palmitic acid and EPA display opposing effects on actin polymerization. As a result, palmitic acid and EPA alter melanin content in melanoma by modulating actin polymerization, thereby affecting melanosome trafficking. Concurrently, DHA interacts with the receptor for activated C kinase 1 (RACK1), thus repressing melanoma cell proliferation through the suppression of protein kinase C (PKC) signaling [338]. The anticancer effect of ω-3 PUFAs is ascribed to their ability to downregulate pro-inflammatory eicosanoid synthesis from COX-2 [320].

Nikolakopoulou et al. [339] tested the effectiveness of DHA and EPA on pre-malignant keratinocyte growth. Both ω-3 fatty acids inhibited HaCaT cell growth. Therefore, a potential combination of anti-cancer drugs with PUFAs could be advantageous and synergistically inhibit carcinogenesis. Moreover, quercetin fatty acid esters like quercetin linoleic acid and ALA positively affected the apoptosis, mechanical properties, and ERK expression in the A375 melanoma cell line. A half-maximal inhibitory concentration (IC_50_) of 35 μg/mL provided amplified A375 cell viability, dose-dependently, as well as the highest cell proliferation inhibition with improved elastic modulus and cell–cell adhesion forces (253 ± 11.2). A notable decrease in phosphorylated ERK levels (0.1439) and apoptosis in A375 cells were also observed [340].

#### 4.2.4. Fatty Acids as Anti-Dermatitis and Anti-Erythema Cosmetic Agents

Dermatitis is a skin disorder characterized by inflammation and itching, often with a predilection for cutaneous flexure [341]. Symptoms include intense itching, erythematous papules with excoriation, vesicles over erythematous skin, thicker skin plaques, enhanced skin marking (lichenification), and fibrotic papules (*Prurigo nodularis*) [342]. Possible outcomes of dermatitis involve defects in barrier function, which can lead to bacterial and allergen invasion, as well as transepidermal water loss and fat loss [343]. Various types of dermatitis, such as atopic dermatitis, discoid eczema, irritating contact dermatitis, seborrheic dermatitis, frictional lichenoid dermatitis, and allergic contact dermatitis, are categorized following diagnosis based on established criteria. Fish oil and its associated fatty acids have been suggested to be beneficial in reducing dermatitis symptoms [343].

A recent clinical trial revealed that the use of ω-3 fatty acid cream in irritant contact dermatitis patients notably increased skin hydration (by reducing transepidermal water loss), with no adverse side effects [344]. Interestingly, cotton shirts with γ-linoleic acid-enriched borage oil reduced erythema, pruritus, and TEWL in the back of patients with moderate-to-severe atopic dermatitis (AD). This study revealed that AD may be ameliorated by external application to the skin, setting the basis for the development of soothing, anti-inflammatory cosmetics [321]. Recent “Dermatologica” study outcomes exhibited the significance of ω-3 PUFAs for improving skin outcomes following 28 days of microneedling in adults with healthy skin, as compared to an HA serum. ω-3 PUFAs function by accelerating inflammation resolution and wound-healing, offering improved redness, brightness, skin texture, erythema, and luminosity [345].

A novel ointment formulation comprising 30% petroleum jelly, 10% cod liver oil, beeswax, sunflower oil, sweet almond oil, and butylated hydroxytoluene (BHT), free of antibiotics and corticosteroids, revealed vast therapeutic efficacy, tolerance, and safety. Patients experiencing recessive dystrophic epidermolysis bullosa (RDEB), reported pain absence, increased sleep, reduced analgesic use and affected areas, rapid blister healing, and notable enhancement in their quality of life [346].

#### 4.2.5. Fatty Acid Agents Against Psoriasis and Acne Vulgaris

Psoriasis is a complex systemic inflammatory disease that damages various tissues and organs due to prolonged immune system activation and an increased release of pro-inflammatory cytokines [347]. This T-cell-mediated skin disease affects 2–3% of the population and is characterized by elevated amounts of circulating and lesional inflammatory T-helper (Th) Th17, Th22, and Th1 cells, which form a complex with IL-17, IL-23, IL-22, and interferon-γ (IFN-γ) cytokines, respectively. Erythematous, scaling lesions are the most common sign of psoriasis, which can present with several clinical phenotypes, including vulgar, inverse, erythrodermic, pustular, and guttate forms [348]. The exact etiology of psoriasis is not yet fully understood, but several risk factors such as obesity, medication, diet, trauma, stress, and smoking have been identified [349]. Patients with psoriasis may also exhibit disrupted lipid and amino acid metabolism, as it is a skin condition characterized by abnormal keratinocyte hyperproliferation, which activates T-cells, leading to the production of arachidonic acid and, ultimately, the generation of multiple pro-inflammatory mediators [350].

Tretinoin–fatty acid (oleic acid) vesicles are prominent anti-psoriatic topical delivery systems that notably increase skin absorption and retention compared to a tretinoin solution alone. Preclinically, significant in vivo anti-psoriatic properties were observed in a mouse model, regarding well-demarcated papules, erythema, and reduced epidermal thickness, whereas reduced spleen weight and IL-6/17 cytokine levels were confirmed as well. Thus, tretinoin–oleic acid vesicles elevated the water solubility and skin permeability of tretinoin and its anti-psoriasis activity [351]. Furthermore, supplementary treatment with ω-3 PUFAs complements topical treatment in psoriasis, notably contributing to reduced psoriasis area and severity index (PASI) and nail psoriasis severity index (NAPSI) and enhanced dermatology life quality index (DLQI). As a result, decreased scalp lesions and pruritus, erythema, scaling, and infiltration of the treated areas were reported [352].

Acne vulgaris is another chronic inflammatory condition, predominantly affecting pilosebaceous follicles. Approximately 85% of teenagers are likely to develop acne, but this dermatosis can begin in childhood and persist beyond adolescence. Clinically, it is characterized by inflammatory lesions (papules, pustules, or cysts) and open and closed comedones, which can be present on the face, chest, and back regions, areas rich in sebaceous glands [353,354]. Interestingly, Jung et al. [355] hypothesized an association between acne and diet, specifically finding that Koreans who consumed less fish presented with acne more frequently compared to healthy patients. Conversely, Landro et al. [356] studied Italians and concluded that those who consumed more fish were shielded against moderate and severe acne [357].

Plant and especially marine phytochemical compounds could be great treatment agents against acne vulgaris. Marine sponges and coral powders hold great potential as natural sources of marine-derived cosmeceuticals for acne prevention, protecting against UV radiation, while enhancing skin rejuvenation [78]. Balboa et al. [358] isolated a tetraprenyltoluquinol meroterpenoid from *Sargassum muticum* and revealed that this terpenoid could attenuate UV_A_-induced damage in vitro. It also protected against ROS production, displaying an important antioxidant activity comparable to retinoic acid [2]. Similarly, *Asparagopsis armata* (commonly known as harpoon weed) served as an effective natural ingredient in skincare formulations, being widely effective in the battle against acne vulgaris. Its fatty acid composition was linked to its antimicrobial potential, whereas glycerolipids enhanced its antioxidant benefits [359].

#### 4.2.6. Fatty Acids as Anti-Inflammatory Cosmetic Agents

Marine ω-3 PUFAs, primarily DHA and EPA, are well-known immunomodulatory and anti-inflammatory substances that can influence the inflammatory process through various mechanisms [360]. Specifically, ω-3 PUFAs feature a double bond between carbons 3 and 4, with DHA being found in higher amounts in fresh lean and oily fish [361]. They exert different biological actions via three main methods: (1) serving as substrates for the biosynthesis of specialized pro-resolving mediators (SPMs), (2) incorporating into cell membrane phospholipids, and (3) acting as agonists of cellular receptors [362].

The anti-inflammatory activity of ω-3 PUFAs is primarily achieved through their incorporation into cell membrane phospholipids, where arachidonic acid (ARA) is also present. This incorporation decreases the amount of AA in the membrane, consequently inhibiting AA metabolism and the expression of COX genes. This process leads to a reduced production of AA-originated eicosanoids [362]. Inflammatory stimuli activate these processes, subsequently activating the phospholipase A2 enzyme. This enzyme is responsible for the hydrolysis of the sn-2 chain of glycerol phospholipids, resulting in the generation of free AA, EPA, or DHA. AA, an ω-6 PUFA, is typically esterified at the sn-2 position of membrane phospholipids. Three primary enzymatic pathways—COX (COX1 and COX2), lipoxygenase (LOX, 5-LOX, 12-LOX, and 15-LOX), and cytochrome 450 mixed-function oxidase enzymes (CYP450)—are in charge of producing eicosanoids from AA. The most well-known mediators and regulators of inflammation are the classical eicosanoids, prostaglandins (PGs), thromboxanes (TXs), and leukotrienes (LTs) [362].

In this context, the in vivo topical application of combined sardine extract (rich in DHA and EPA) with ketoprofen notably increased skin penetration compared to extracts from mackerel and horse mackerel. This combination also inhibited UV_B_-induced erythema by 60.5%, whereas sardine oil extract alone inhibited it by 24.5%, and ketoprofen alone by 46.6% [320]. The anti-inflammatory potential of marine fatty acids is well-documented, supporting their incorporation into creams, emulsions, cosmetic masks, lipsticks, bath fluids, and nail polishes. Moreover, ethyl oleate extracted from a symbiont of the marine sponge *Dendrilla nigra* has displayed substantial anti-inflammatory activity, indicating its promise as a multifaceted cosmeceutical in skincare formulations [78]. Furthermore, free fatty acids derived from cod liver oil have shown great potential in enhancing drug permeation through the skin and oral mucosa. These fatty acids are less toxic and have shown no adverse effects, suggesting their suitability as active ingredients in various cosmetic applications [363].

### 4.3. Marine Polar Lipids and Lipid Vitamins as Cosmetic Ingredients

Marine-derived polar lipids and lipid-soluble vitamins are emerging as high-value bioactives in cosmetic science due to their multifaceted roles in promoting skin health, barrier protection, and anti-aging benefits. Marine organisms, including microalgae, krill, sea cucumbers, mollusks, crustaceans, and fish by-products, are rich sources of unique polar lipids such as phospholipids, glycolipids, sphingolipids, and sulfolipids, which display enhanced bioactivity compared to their terrestrial counterparts due to their high PUFA content. Marine phospholipids (rich in EPA and DHA) are integrated into skin lipids, offering antioxidant, anti-inflammatory, and hydrating effects [343,364,365].

These polar lipids also play a critical role in reinforcing the stratum corneum lipid matrix, enhancing moisture retention, and protecting against TEWL, a key feature in treating dry and aged skin. Marine sphingolipids and glycolipids extracted from several marine species (e.g., diatoms, cyanobacteria) also demonstrate skin barrier repair action and possess natural UV-absorbing properties, making them valuable in photoprotective formulations. Polar lipids may offer several benefits as solubilizing vesicles, wetting agents for dispersing lipophilic compounds, accelerating skin hydration and moisture, reducing skin roughness, increasing skin softness and whitening effects, and suppressing several skin-related conditions (e.g., psoriasis, AD, erythema). Moreover, their ability to form liposomes or nanocarriers enhances the delivery and bioavailability of other bioactives in cosmetic products [78,364,365,366].

Marine phospholipids in the cosmetic industry are typically utilized as emulsifiers in natural cosmetics. For example, self-emulsifying, high-internal-phase emulsions using endogenous phospholipids from Antarctic krill oil, in addition to their significant value in functional food production, may also serve as functional cosmetic ingredients [367]. Hydrophilic compounds (e.g., lecithin) can also be encapsulated by marine polar lipid-based liposomes, which may act as solubilizers, wetting agents, moisturizers, and penetration enhancers in cosmetic preparations. Marinosomes, a natural mixture of marine lipids high in PUFA (e.g., EPA, DHA), showed great stability mimicking topical application conditions, reduced inflammatory responses (e.g., PGE2, IL-8), and could potentially act as an antioxidant and anti-inflammatory agent against UV-induced skin complications [366,368,369,370]. Furthermore, lecithin extracted from rainbow trout fish, eggs, and other marine organisms has displayed notable emulsifying, moisturizing, and skin-penetrating benefits, better delivery, and long-term stability in cosmetics [371,372].

Simultaneously, marine sources provide a rich supply of lipid-soluble vitamins, mainly vitamin A, D, E, and K, either directly from marine organisms or indirectly through marine oils and algae-derived products. For instance, vitamin A analogs, such as retinol and retinaldehyde, extracted from fish liver oils, are widely used to stimulate collagen synthesis, reduce fine lines, and normalize keratinocyte turnover [320,373,374,375]. Marine vitamin D_3_ (cholecalciferol), primarily derived from fish oils, contributes to cellular differentiation and may alleviate hyperproliferative conditions like psoriasis or seborrheic dermatitis [376,377,378]. Marine-derived tocopherols and tocotrienols, collectively referred to as vitamin E compounds, exert strong antioxidant and photoprotective effects, quenching ROS and reducing UV-induced erythema and photo-aging markers [78,379]. Additionally, vitamins K1 and K2, found in marine algae and fermented seafood, may support vascular health and decrease periorbital dark circles or couperose by improving microcirculation and capillary stability [380,381].

Crucially, both marine polar lipids and lipid-soluble vitamins can be integrated into advanced delivery systems, such as marine phospholipid-based liposomes, solid lipid nanoparticles (SLNs), and nanoemulsions, enhancing stability, cutaneous penetration, and targeted release. These innovations not only improve the efficacy of the bioactives but also align with current trends favoring natural, sustainable, marine-derived cosmetic and cosmeceutical products [78,366].

### 4.4. Marine Amino Acids as Cosmetic Ingredients

Amino acids serve as the building blocks of proteins, including collagen, elastin, and keratin, which are essential for maintaining skin structure and function. An unbalanced amino acid ratio can reduce the synthesis of these structural proteins, leading to sagging, wrinkles, skin thinning, dryness, and loss of elasticity [382,383]. Marine sources are valuable reservoirs of amino acids, particularly relevant for skin health. For instance, glycine, proline, and hydroxyproline are critical for collagen stability, while lysine is essential for crosslinking collagen fibers [384]. Deficiency in these amino acids impairs collagen formation, increasing the risk of wrinkles and delaying wound healing. Moreover, amino acids like arginine and histidine are known to enhance skin hydration and barrier repair, with arginine also stimulating nitric oxide production, which improves microcirculation [384,385].

Malfunction or insufficient production of these proteins may result in conditions such as urticaria, eczema, thrush, xerosis, itching, and skin ulcers. Furthermore, epidermal keratinocytes require amino acids to synthesize certain antimicrobial peptides (e.g., cathelicidins and defensins), which combat pathogens and maintain a balanced skin microbiome [382,383]. Commercially, several amino acid-based skincare products are currently available. For example, mycosporine-like amino acids (MAAs), as natural UV-absorbing compounds, protect against UV exposure, improve skin elasticity, and reduce fine lines, sunburn, and therefore, photo-aging [18,386].

Amino acid replacement is essential in the skin, particularly during stratum corneum shedding. This requirement for specific amino acids is linked to two factors: (1) their relative abundance in the primary skin protein sequence and (2) their film-forming capability. Traditionally, amino acids are classified as essential and non-essential, as human cells cannot synthesize all proteinogenic amino acids de novo; hence, humans must obtain non-essential ones through diet or supplements. Both groups are vital for skincare [387].

However, beyond general nutritional importance, marine-derived amino acids have emerged as targeted cosmeceutical ingredients with specific skin benefits. In practice, each of the 20 standard amino acids, derived from various marine sources, contributes to the creation and maintenance of smooth, healthy, and youthful-looking skin while also regulating the aging process. They are safe, nontoxic, sustainable, and natural sources that do not trigger allergic reactions, possessing antioxidant effects and promoting collagen production [382]. For example, taurine sourced from marine algae and fish has been shown to improve skin hydration and strengthen the skin barrier by regulating osmolyte balance and protecting against dehydration, making it valuable in anti-aging skincare [388].

#### Mycosporine-like Amino Acids (MAAs)

The most prevalent class of secondary metabolites contained in aquatic species is mycosporine-like amino acids (MAAs). They are present in a wide range of marine and freshwater organisms, including cyanobacteria, fungi, algae, and higher-order creatures, such as cnidaria, fish, arthropods, tunicates, echinoderms, and mollusks. MAAs find application in cosmetics as UV-protecting agents because they can absorb UV_A_ (315–400 nm) and UV_B_ (280–315 nm) radiation without producing free radicals. The majority of different MAAs absorb within the UV_A_ range. Examples involve mycosporine-2-glycine, shinorine, and porphyra-334, all having a λ_max_ between 332 and 334 nm. Palythine, palythine–serine, and palythine–threonine have a λ_max_ of 320 nm, while palythine–serine sulfate and palythine–threonine sulfate have a λ_max_ at 321 nm. At a higher UV_A_ wavelength range (357–362 nm), usujirene, palythene, and euhalothece-362 can absorb [389]. Significant amounts of MAAs have been reported in sponges [390] and fish [391,392]. The accepted concentration of MAAs for use in sunscreens or essences is 0.001–15.0 and 0.01–2.0% (by weight), respectively [393]. Therefore, these concentrations align with the accepted ingredient concentrations for UV protection in sunscreens, which typically range from 1 to 5% (by weight) [394].

MAAs have displayed notable anti-photo-aging, antioxidant, and anti-UV radiation photoprotective properties. Rosic et al. [389] conducted a review on the use of MAAs as molecules capable of providing skin protection. MAAs demonstrated antioxidant function and could prevent damage from singlet oxygen via scavenging ROS. Their work also revealed that over 30 species of MAAs exist in nature, differing in their antioxidative and UV-absorbing capabilities.

Many studies have additionally shown that MAAs may be useful as antioxidants [395,396,397]. Various MAAs can scavenge ROS, like hydroxyl radicals, hydroperoxyl radicals, singlet oxygen, and superoxide anions. MAAs’ antioxidant function may be especially important in scavenging free radicals disseminated by oxidative stress caused by UV light or other environmental stimuli. Among the 30 currently known MAAs, several mono-, di-, and glycosylated MAAs have exhibited antioxidant activity in both in vitro and in vivo studies [395,396,397]. It should be mentioned that certain MAAs also showed indirect evidence of antioxidant activity through a reaction with singlet oxygen or a gradual photo-degradation in the presence of a photosynthesizer. Some in vitro studies have indicated that abiotic stressors such as temperature, desiccation, acidity, and salinity may significantly enhance MAAs’ antioxidative capabilities [398].

Regarding their anti-UV effects, Sirisattha et al. [399] studied the efficiency of MAA-containing emulsion in filtering UV_A_ and longer UV_B_ wavelengths. The test, carried out on mouse ear tissue exposed to UV irradiation, showed that the MAA emulsion increased the activity of both total superoxide dismutase (SOD) and catalase (CAT). Thus, the MAA emulsion protects antioxidant proteins and therefore upregulates antioxidative processes [396]. Table 5 presents examples of skin-related cosmetic patents comprising MAAs.

### 4.5. Predominant Marine Proteins as Cosmetic Ingredients

#### 4.5.1. Marine Collagen as a Cosmetic Agent

Collagen stands out as a highly beneficial beauty ingredient, distinguished by its excellent biocompatibility and minimal immunogenicity within the human body, making it an ideal component for cosmetic formulations. Type I collagen is particularly favored in cosmetic products because its composition mirrors that found in human skin, and it is also abundant in fish scales, swim bladders, and bones [410]. Given that commercial collagen from land-based sources has been linked to zoonotic diseases, marine collagen serves as a crucial alternative to collagen derived from pigs and cows [46]. Additionally, concerns from the Muslim community regarding halal cosmetics, which prohibit the use of gelatin and bioactives from porcine sources (plus bovine sources under certain conditions [411]), further highlight the significant future potential of marine-originated collagen.

Collagen extracted from marine fish is extensively used in cosmeceuticals because of its beneficial bioactive properties for skin regeneration and healing. Compared to collagen generated from animal sources, marine fish-derived collagen exhibits superior absorption capabilities [412]. Furthermore, marine collagen offers many advantages, such as a mild odor and good mechanical strength, which are basic requirements for its cosmetic use [413]. Cosmetic formulations (cream or serum) containing fish-derived collagen have been tested for their ability to hydrate and tighten the skin. Results indicated that serum formulations provided quicker hydration [414]. Over time, particularly after repeated applications, cream formulations appeared to become more active. Nevertheless, both lotion and cream formulations produced a long-lasting tensor (firming) effect throughout the treatment period [414].

Type I collagen is the most common type used in collagen-containing cosmetics, demonstrating anti-aging [415,416], anti-wrinkle [415], moisturizing, antioxidant, skin whitening, and UV-protection [417] activities. Collagen possesses the ability to form films that prevent TEWL, making it advantageous in both skin and hair formulations [410]. Hydrolyzed collagen is preferred over high MW collagen in cosmetic formulations, due to its superior solubility at neutral pH, ease of dermal penetration, and water-binding properties [418]. Collagen-derived cosmetics can also be used in children’s formulations to prevent irritation caused by anionic surfactants [18,419].

##### Collagen as a Moisturizing Agent

Alves et al. [46] investigated the beneficial effects of marine-originated collagen as a natural humectant and moisturizer, specifically using collagen extracted from salmon and codfish skin. After extensive characterization, results indicated high purity of isolated collagen type I, though its structural and chemical properties varied depending on the source. No irritation or inflammation was observed, affirming its suitability for inclusion in the cosmetic field. Moreover, Jimbo et al. [420] demonstrated that even a low dose of collagen hydrolysate could effectively improve skin hydration by reducing TEWL. Hou et al. [421] evaluated the effectiveness of collagen polypeptides derived from cod fish in UV-induced skin damage. Their study showed that these collagen polypeptides possessed excellent moisture absorption and retention benefits. Their mechanism of action involves reducing moisture and lipid loss, promoting antioxidant properties, repairing endogenous collagen and elastin protein fibers, and also maintaining the ratio between type III and type I collagen [223].

##### Collagen as an Anti-Aging Agent

Reduced collagen content in the skin is an underlying cause of age-related skin thinning. In vivo studies show that type I collagen is upregulated when collagen peptide and vitamin C are administered together. Shibuya et al. observed a decrease in superoxide dismutase 1 (Sod-1) when collagen peptides were supplemented with vitamin C [422]. Subsequent in vitro studies verified that collagen oligopeptide, a digested form of ingested collagen peptide, enhanced the bioactivity of vitamin C derivatives concerning fibroblast migration and proliferation [422]. The combination of collagen peptide and vitamin C derivative also increased the skin thickness of hairless Sod-1-deficient mice. Rahman et al. [423] discovered the anti-aging properties of a supplement containing marine fish collagen enriched with essential amino acids, such as proline and glycine, affirming its potential to mitigate the aging process.

##### Collagen as a Wound-Healing Agent

Elbialy et al. [424] examined whether collagen from Nile tilapia (*Oreochromis niloticus* L.) could promote wound healing in rats. Their study found that adding tilapia collagen to a wound accelerated healing by enhancing keratinocyte proliferation, fibroblast and myofibroblast differentiation, and ECM synthesis. Similarly, Shalaby et al. [425], studied the effects of collagen from grey mullet and tilapia on rat wound healing. Their findings indicated that improving cell adhesion capabilities promotes wound healing, which in turn enhances wound closure and resolution. Furthermore, the concentration of hydroxyproline, a specific component of the collagen protein, can be used as a tool to estimate collagen deposition and, consequently, the effectiveness of wound healing. Generally, many marine-based studies have proven marine organisms’ collagen potential in scaffolds destined for skin tissue regeneration. For instance, a composite film made from salmon mint DNA and collagen, modulated would regeneration and tissue engineering in mice [412].

#### 4.5.2. Marine Gelatin as a Cosmetic Agent

Gelatin obtained from marine fish by-products has exhibited greater antioxidant activity compared to synthetic gelatin [178]. Many cosmetic products, including face creams, body lotions, shampoos, hair sprays, sunscreens, and bath salts and bubbles, utilize gelatin as a gelling agent. Proteins and peptides found in fish gelatin hydrolysates have been employed to mitigate UV-ray damage to the skin. By preserving the skin’s balanced lipids through their antioxidant qualities, they contribute to the repair of damaged skin structure [412,426].

Chen et al. [427] investigated the protective effects of gelatin hydrolysate obtained from the Pacific cod (*Gadus macrocephalus*) against UV-radiation-induced inflammation and collagen reduction in photo-aging mouse skin. Oral administration of gelatin hydrolysate deactivated UV-radiation-induced inflammation by inhibiting endogenous antioxidant enzyme activity and suppressing the expression of NF-κB, as well as NF-κB-mediated expression of pro-inflammatory cytokines. Additionally, by upregulating the type II transforming growth factor β (TGF-β) receptor (TβRII) level and downregulating Smad7 levels, gelatin hydrolysate inhibited the synthesis of type I procollagen. This indicates that gelatin hydrolysate is involved in matrix collagen synthesis by activating the TGF-β/Smad pathway in the photo-aging skin.

Recent studies have focused on the photo-aging effects of gelatin and its hydrolysate extracted from salmon skin [428]. Gelatin and its hydrolysates displayed an average molecular weight of 65 kDa and 873 kDa, respectively [428]. Furthermore, tilapia gelatin peptides were studied concerning UV-induced skin damage in mice [429]. The findings suggested that tilapia gelatin could prevent UV-ray damage by shielding the skin’s lipids and collagen. The tilapia gelatin peptides were found to contain the antioxidant peptide Leu-Ser-Gly-Tyr-Gly-Pro (592.26 Da), which has an IC_50_ value of 22.47 µg/m and can scavenge hydroxyl radicals [429,430].

### 4.6. Peptides as Cosmetic Ingredients

In skincare, the most important amino acids include the three cationic ones, namely histidine, lysine, and arginine, and the three neutral ones, namely glycine, proline, and leucine, with the latter being abundant in collagen. Beyond their necessity for protein synthesis, amino acids also perform specific functions vital for maintaining skin health. Since they are complementary, no single amino acid can be considered the most significant. Lee et al. [431] demonstrated that the combination of lysine and arginine accelerated the wound-healing process, increased both biocompatibility and hydrophilicity, and could prevent infections. In another study, the combination of leucine with glycine and proline was proved beneficial in improving skin firmness and wrinkles [432]. Examples of amino acid peptide chains and their skin-related benefits are presented in the following table, Table 6.

Marine proteins are comprised of small peptides, which are inactive fragments of complete protein sequences. Bioactive peptides are extracted from marine organisms and waste via enzymatic hydrolysis. Tuna, salmon, and eels are notably rich in histidine-containing dipeptides, including carnosine (β-alanylhistidine) and anserine (β-alanyl-1-methylhistidine). These peptides are vital components in cosmetic and cosmeceutical fields. Typically, bioactive peptides consist of 3–20 amino acid residues. Marine peptides exhibit multiple effects, including immunostimulant [446], antibacterial [446], antioxidant [383], anti-photo-aging [447], anti-aging [448], and anti-cancer [449,450]. Alcalase, α-chymotrypsin, papain, pepsin, neutrase, and trypsin are the most frequently used proteinases for the hydrolysis of fish proteins [451].

Marine-derived peptides have exhibited anti-radical and antioxidant effects used for anti-aging and photoprotective applications, tyrosinase-inhibiting effects with whitening cosmeceutical abilities, MMP-inhibiting effects employed in anti-wrinkle formulations, and anti-inflammatory and antimicrobial benefits applied in cosmetic skin soothing products. For instance, vast photoprotective and anti-photo-aging peptides have been extracted from fish, like collagen polypeptides and gelatin hydrolysate from cod skin, gelatin peptides from tilapia, and marine collagen peptides from *Pollachius virens*, *Hippoglossus hippoglossus*, and *Pleuronectes platessa* [383,412,452].

Peng et al. [447] conducted a study on the identification and purification of peptides from oyster (*Crassostrea hongkongensis*) protein enzymatic hydrolysates that possessed potential anti-photo-aging effects. Two peptides were identified, WNLNP (Try-Asp-Leu-Asn-Pro) and RKNEVLGK (Arg-Lys-Asn-Glu-Val-Leu-Gly-Lys). The first peptide, WNLNP, demonstrated dose-dependent anti-photo-aging effects, while the second, RKNEVLGK, exhibited protective properties by inhibiting ROS production, decreasing MMP-1 expression, and increasing extracellular pro-collagen I. Through multiple analyses, the pentapeptide WNLNP had a higher potential in preventing and regulating the skin photo-aging process [447]. Furthermore, Lu et al. [453] showed that the peptides derived from cod skin gelatin hydrolysates effectively inhibited phosphorylated-*p38* and ERK in the MAPK pathway, as well as MMP-1, and therefore served as skin-protecting supplements. Finally, Hu et al. [449] investigated a polypeptide fraction from the bivalve mollusk *A. Subcrenata*, both in vivo and in vitro. The results showed significant inhibition against HeLa and HT-29 tumor cell lines, as well as a reported antioxidant effect due to the presence of an unidentified polypeptide, which had a molecular weight of 20,419 Da. Marine peptides with anti-aging, skin-protective, and other health-promoting properties are included in Table 7.

### 4.7. Marine Pigments as Cosmetic Ingredients

The market demand for natural carotenoids is reportedly increasing, bringing up the necessity to improve their production from natural and renewable sources as an alternative to synthetic products [465]. Indeed, while algae dominate the marine pigment section, several non-algal marine (micro) organisms, together with fish, crustaceans, and mollusks, produce or accumulate pigments with notable cosmetic and cosmeceutical potential. For example, astaxanthin, zeaxanthin, lutein B, tunaxanthin, halocynthiaxanthin, fucoxanthin, and β-carotene from the Japanese mackerel (*Scomber japonicus*/*Pneumatophorus japonicus*), rainbow trout (*Oncorhynchus mykiss*), and Japanese amberjack/yellowtail (*Seriola quinqueradiata*) have shown vast antioxidant, anti-UV, and radical scavenging potential for integration in cosmetics and cosmeceuticals [11].

Fish by-product carotenoids are extensively used as preservatives in cosmetics, together with other antioxidants or algal bioactives, as well as in creams and lotions offering sun protection [192,466]. Astaxanthin and melanin are marine pigments extracted from fish, crustaceans, and mollusk sources, and have been widely applied in cosmetics and cosmeceuticals [465].

#### 4.7.1. Astaxanthin as a Cosmetic Agent

Astaxanthin (AST), predominantly found in crustacean shells, is recognized as the most important carotenoid. Various extraction techniques, including organic solvent, SFE, and MAE, can be employed to obtain AST from crustacean carapaces [28,467]. Its skin benefits include antioxidant and anti-aging [468,469], moisturizing [470], anti-wrinkle [469], UV-ray-protecting [469], anti-tumor [471], anti-eczema, and wound-healing properties [472]. AST is found in high concentrations in shrimp head, shell, and tail [473].

In crustaceans, AST plays a crucial role as an immune-stimulating and antioxidant agent. Its immune-stimulating function is attributed to the presence of peroxinectins, proteins involved in immune defense mechanisms, and peroxidase activity. Regarding its antioxidant activity, crustaceans can scavenge free radicals through both endogenous enzymes and non-enzymatic exogenous compounds. High levels of irradiation (primarily UV), oxygenic photoautotrophy, and the presence of xenobiotics can all lead to the production of ROS. SOD, CAT, and GSH-P_X_ are the main antioxidant enzymes capable of eliminating ROS (anti-radical activity). Due to AST’s low water solubility, limited chemical stability, and poor oral bioavailability, there is a need for alternative delivery methods to incorporate it into cosmetic and pharmaceutical formulations [113].

Several astaxanthin delivery systems have already been found efficacious towards skin-related functions and cosmetic/cosmeceutical applications. Vesicular AST micro- and nano-emulsions revealed increased solubility and bioavailability following topical absorption; however, liposomes show reduced vesicle flexibility, and topical application requires reaching the stratum corneum, which is challenging [474,475,476]. Moreover, AST micro- and nano-emulsions act as effective delivery systems for rapid penetration, long-lasting effects, and uniform skin deposition [474,477], while particulate AST delivery systems like micro- and nanoparticles create compatible environments for susceptible molecules, increasing stability against degradation and controlled, targeted release [474,478]. Finally, AST inclusion complexes (AST cyclodextrins (CDs)) are suitable for the topical delivery of poorly soluble compounds, whereas AST polymeric films or hydrogels have demonstrated excellent skin adherence and evaporation after contact, but supporting research is yet limited [474,479,480].

AST has gained widespread acceptance as an antioxidant in skincare formulations [465]. For example, Niu et al. [481] suggested that AST’s mechanism involves inducing the production of ROS in cells rather than directly scavenging free radicals. This small amount of ROS is generated after the activation of the Nrf-2/heme oxygenase 1 (HO-1) antioxidant pathway. When compared to a control group, dietary AST supplementation dramatically reduced wrinkle formation and TEWL, while also maintaining epidermal barrier functions in the dorsal skin of HR-1 hairless mice exposed to UV_A_ [482]. Moreover, shrimp (*L. vannamei*) AST showed notable ROS and singlet-oxygen-scavenging ability, as well as tyrosinase inhibition, suggesting its skin-health-promoting effects [36].

Regarding its wound-healing value, Fang et al. [483] investigated the prevention of early burn-wound progression using AST. A rat deep-burn model was employed to study the effects of AST on the skin through histological and biological assessments. The results demonstrated that this natural antioxidant offers protection against burn wounds by mitigating oxidative stress-induced inflammation (inducible nitric oxide synthase (iNOS)-induced) and accelerating wound healing [484]. Additionally, the moisturizing properties of AST were reported by Ikarashi et al. [470]. They showed that it can improve water channel aquaporin-3 (AQP-3) activity and expression in the skin, thereby increasing skin moisture. In their study, when AST was added to PHK16-0b or HaCaT cells, mRNA expression levels of AQP-3 increased in a concentration-dependent pattern in both cell lines. AQP3 expression also increased when AST was added to HaCaT cells. In the EpiSkin model, AST addition increased the AQP-3 expression. The combination of glycerol with AST in the EpiSkin model increased glycerol permeability compared to glycerol alone. This study suggests that AST is an important candidate for treating dry skin, particularly in cases of diabetes mellitus and aging [470].

Carotenoids from fish, mollusks, or crustacean by-products, including AST, serve as preservatives in cosmetics, often combined with other antioxidants or algal bioactives in creams and lotions for sun protection [192,466]. Ito et al. [485] studied AST’s protective effects against UV rays. A 10-week double-blind, placebo-controlled study involving 23 healthy Japanese participants was conducted. To demonstrate AST’s protective effects, the minimal erythema dose (MED) and UV-induced skin damage regarding moisture content and TEWL were measured after 9 weeks of supplement use. The results showed increased MED and decreased skin moisture loss in the affected area compared to the placebo. Moreover, improvement in rough skin and texture was observed in areas not exposed to irritation. Its anti-UV skin damage benefits were also reported by Dutta et al. [486], who proposed AST use in sunscreens, highlighting its role as a natural, biodegradable UV-filter with great protective effects against aging, inflammation, and cancer. Finally, according to Seki et al., krill-based topical AST reduced erythema by ~60% within 4 days post-UV_B_ exposure in seven male subjects, while Tominaga et al., supported that in 28 women, a combined use of krill-based oral supplements and topical cream for eight weeks led to notable wrinkle-depth reduction and skin elasticity improvement [487].

AST also has the potential to inhibit the production of inflammatory mediators, thus exerting anti-inflammatory effects [482]. Older individuals, due to slowed metabolic processes leading to weakened skin barriers, are more susceptible to atopic dermatitis (AD) [488]. An AST cream could be used to prevent phthalic anhydride (PA)-induced AD in HR-1 hairless mice by inhibiting the release of various inflammatory cytokines [488]. Specifically, topical application to the mice’s ears or back skin reduced inflammation and hyperkeratosis. These effects are mainly linked to the downregulation of NF-κΒ and its pro-inflammatory genes COX-2 and iNOS [489,490]. Lee Y.S. et al. [491] studied the anti-inflammatory effects of liposomal AST on a PA-induced AD model. L-AST showed positive effects on PA-induced dermatitis severity, mast cell infiltration, and epidermal thickening. It also suppressed the activation of inflammatory mediators and markers. Consequently, the results indicated that L-AST was more effective than free AST due to its inhibition of oxidative stress, signal transducer and activator of transcription 3 (STAT3), and NF-κΒ signaling pathways, while also achieving a reduction in immunoglobulin E (IgE), a marker associated with allergic reactions.

In a comparative study, Shanmugapriya et al. [492] used an experimental design for optimization to create homogenous and stable oil/water nanoemulsions containing AST or α-tocopherol, employing spontaneous and ultrasonication emulsification methods. The anti-cancer, wound-healing, and antibacterial properties of the AST-containing nanoemulsions suggest their potential application in formulations for the treatment of skin cancer and wound-healing, such as via inclusion into films. At this point, several AST-based cosmeceuticals have also offered significant skin-associated benefits. The following table, Table 8, describes some clinical trials using AST cosmeceuticals, confirming AST’s importance in cosmetic formulations.

#### 4.7.2. Melanin as a Cosmetic Agent

The cosmetics industry increasingly prioritizes the discovery and utilization of natural active ingredients. Melanin, a natural nanocomponent, contains several active chemicals [17]. Firstly, as a nontoxic natural pigment, melanin serves as an effective natural colorant in cosmetics. Secondly, its antioxidant and anti-UV properties allow it to function as a photoprotective agent in cosmetics, thereby extending the anti-aging period of such products. Additionally, similarly to vitamins C and E, melanin exerts an opsonizing effect on the skin, acting as an anti-aging agent [495]. Squid ink melanin, marketed as “Creanatural^®^ Sepia Melanin”, is a cosmetic ingredient incorporated into skin, hair, and sun-care products [78,496].

Melanin plays a crucial role as a UV filler due to its significant impact on epidermal homeostasis, which is closely linked to melanocyte behavior. Melanin protects the skin from UV rays by absorbing them and releasing the absorbed energy as heat, effectively preventing direct UV radiation from reaching the DNA of the epidermal cells [497]. In more detail, melanin scavenges ROS, which are generated in the skin during UV-induced oxidative stress. The amount of melanin present in the skin is determined by its phototype. However, in most phenotypes, the natural melanin levels are insufficient to provide adequate UV protection during the summertime. Consequently, photoprotective molecules should be integrated into sunscreens [78,498,499].

Interestingly, melanins, including allomelanin, neuromelanin, eumelanin, pheomelanin, and pyomelanin, found in cephalopod ink, marine bacteria, and fungi, have offered significant anti-inflammatory and antioxidant cosmetic benefits [78]. Finally, cephalopod (*Sepia officinalis*) ink has been incorporated into mascara, eyeshadow, and other cosmetic products. These products displayed very satisfactory outcomes in terms of the level of aspect, texture, color, and covering capacity of the formulated cosmetic products [91,500].

### 4.8. Phenolic Compounds as Cosmetic Ingredients

Phenolic compounds are found in high amounts in marine organisms. Phenolics include bromophenolic compounds, simple phenolic acids (e.g., hydroxycinnamic acids, hydroxybenzoic acids), flavonoids, and phlorotannins [501]. Among these, flavonoids appear to be the most studied group, comprising compounds such as quercetin, rutin, and catechins, which are predominantly found in algae and fish [502]. Numerous cyanobacteria and macroalgae have been identified as rich sources of bromophenolic compounds. A food chain transfer of these compounds can occur from macroalgae to invertebrate grazers and subsequently to fish. Their characterization is crucial, as some can be toxic [503]. Bromophenols (including hydroxylated and methoxylated bromodiphenyl ethers) are believed to originate from natural sources and the biotransformation of natural and anthropogenic compounds, given the limited reports regarding their industrial application [504]. Natural marine bromophenols are primarily detected in red algae but are transferred to fish, shrimp, and crabs via the food chain. Notably, higher levels of hydroxylated and methoxylated polybrominated diphenyl ethers (PBDEs) have been found in bivalves (clams and mussels), compared to other types of shellfish [504]. Furthermore, finfish exhibit higher concentrations of PBDEs than shellfish, according to Cade et al. [504]. Tribromophenol concentrations in aquatic species range from 7 to 1600 ng/g wet weight (*w*/*w*) in algae, 0.3 to 2360 ng/g *w*/*w* in crustaceans, 0.9 to 198 ng/g dry weight in mollusks, and 3.7 to 230 ng/g *w*/*w* in fish, as compiled by Koch and Sures [505].

Phenolic compounds (PCs) exhibit excellent antioxidant, anti-inflammatory, and anti-cancer effects. Shellfish, including shrimps, clams, and oysters, are particularly rich in marine polyphenols. Their antioxidant and anti-inflammatory activities are attributed to the presence of multiple hydroxyl groups (-OH) [506]. Phlorotannins, including diphlorethol, triphloroethol, trifuhalol, and tetrafuhalol, phloroglucinol, eckol, and eckstolonol from zebrafish and several brown algae species (e.g., *Halidrys siliquosa*, *Ecklonia cava*, *Ascoseira mirabilis*, *Cystosphaera jacquinotii*, and *Ishige okamurae*), have displayed notable in vitro and in vivo antioxidant, UV-protective, and radical-scavenging activities [11]. Moreover, flavonoids, phlorotannins, phloroglucinol, eckstolonol, eckol, phlorofucofuroeckol, and dieckol from zebrafish and marine brown algae (e.g., *Sargassum polycystum*, *Ecklonia stolonifera*, *Ecklonia cava*, and *Sargassum siliquastrum*) also exhibited both in vitro and in vivo interesting tyrosinase inhibition and anti-melanogenesis benefits, suitable for combating hyperpigmentation incidence [11].

Due to the sensitivity of phenolic compounds to environmental factors and processing conditions, new technologies like encapsulation are employed to protect and stabilize them. Encapsulation offers benefits such as targeted release at specific sites, increased bioavailability, and improved bioaccessibility. When encapsulated, marine phenolic extracts also act as a barrier against undesirable odors and flavors that might otherwise transfer to the application matrix [507].

### 4.9. Marine Substrates and Minerals as Cosmetic Ingredients

Despite the widespread use of both synthetic and natural calcium phosphates (CaPs) in cosmetic products such as skincare, haircare, deodorant, and mouth care products, there are a few investigations into fish-derived CaPs as cosmetic ingredients [508]. Recently, lobster-shell-derived calcium significantly proliferated bone and skin cells in vitro; therefore, lobster minerals could serve as a potential source of functional cosmetic and cosmeceutical calcium for commercial products [509]. Teixera et al. [39] and Piccirillo et al. [510] studied the potent properties of hydroxyapatite (HA) derived from cod fish. HA is a calcium phosphate for its very high biocompatibility. The results of both studies showed that HA-Fe_2_O_3_ powder was effective in a wide range of UV wavelengths, and no radicals were produced during irradiation. HA, apparently due to its crystalline nanostructure and relatively low photo-reactivity, may function as a UV-filter that minimizes oxidative damage and irritation. Therefore, a high UV_A/B_ absorption was achieved, making it applicable as a broad-spectrum sunscreen [510].

In another study by Rozaini et al. [511], Fingescale sardinella bones were used to create skin hydrogels containing unmodified HA, Mn-doped HA (HA-Mn), and Fe-doped HA (HA-Fe). These hydrogels displayed a range of sun protection factors, including SPF 20 for HA, SPF 40 for HA-Mn, and SPF 50 for HA-Fe. Because marine-derived CaPs may possess a different trace mineral content and exhibit a different range of action compared to CaPs from terrestrial animals, more research on marine by-product CaPs is required to provide adequate scientific evidence for their application in cosmetics [28]. Marine HA is also currently being explored for its biocompatible skin and teeth remineralization, wound-healing carrier, and skin-rejuvenation properties, as trace elements (e.g., Zn, Mg) derived from HA may promote collagen synthesis, skin cell proliferation, and anti-inflammatory effects [512]. Finally, marine magnesium and zinc, solely, are often added to mineral sunscreens and anti-acne products due to their anti-inflammatory and sebum-modulating properties [78,79].

Pearls, calcified structures that form the inner layer of bivalve cells (comprising aragonite and nacre), have garnered wide interest for cosmetics, primarily in the form of powdered pearl shells or nacreous shell layers. Chiu et al. [513] confirmed that the protein extract of pearl powder exhibited high antioxidant activity and prolonged *C. elegans* longevity, being beneficial for treating several age-related degenerative disorders. Furthermore, mother of pearl (powdered nacre, *Pinctada maxima*) implanted into rat dermis, enhanced their skin tone, regulated skin fibroblast functioning, improved ECM formation, increased tissue regeneration, and thereby served as a highly potential cosmetic agent [514]. Also, powdered pearl shells or nacreous shell layers contain conchiolin, which is known for its skin hydration, collagen-rebuilding, angiogenesis-promoting, and wound-healing cosmetic properties against hyperpigmentation, atopic dermatitis, deep-burn porcine skin, burn scars, and skin cancer incidence [28,514].

### 4.10. Marine Bioactives as Potential Cosmetic Ingredients

As explored in the previous sections, a wide spectrum of marine-derived bioactives, from polysaccharides, lipids, vitamins, and proteins to peptides, amino acids, pigments, phenolics, and minerals, has demonstrated remarkable potential in cosmetic products. Each category presents unique physicochemical properties and biological activities that contribute to skin protection, hydration, repair, and rejuvenation. These bioactives, extracted from diverse marine substrates such as fish by-products, crustaceans, and mollusks, align with the growing demand for natural, sustainable, and multifunctional cosmetic ingredients. To consolidate the extensive information provided, the following table, Table 9, offers an organized overview of the key marine bioactives discussed, their sources, main cosmetic applications, and associated benefits [11,28,78,79].

## 5. Challenges and Future Perspectives

Climate change and global warming are leading to the poleward migration of marine flora and fauna, including macroalgae, mollusks, and fish, altering the geographic distribution of potentially valuable marine resources. As ocean temperatures rise, species previously restricted to tropical and subtropical waters are increasingly found in temperate and even polar regions, potentially expanding the availability of certain marine bioactives, such as fucoidans, PUFAs, pigments (e.g., fucoxanthin, astaxanthin), and peptides, in these new environments. Conversely, warming waters can also reduce the abundance of cold-water species that produce unique bioactives, impacting their qualitative and quantitative supply. Although direct data on temperature effects on bioactive production remain scarce, future marine bioactive sourcing strategies must consider the shifting distribution and biochemical profiles of marine organisms due to global warming, along with adaptive harvesting, cultivation, and bioprocessing methods in polar and temperate regions to harness these changes responsibly. Integrating environmental monitoring with bioactive profiling could enable predictive harvesting and cultivation aligned with sustainability and quality control frameworks, ensuring consistent supply while mitigating climate-driven risks to marine biodiversity and cosmetic raw material security [517,518].

Despite the growing interest and promising evidence regarding the application of marine-derived bioactives in the cosmetic industry, several limitations currently hinder their full-scale utilization. A major challenge lies in the limited scientific documentation and peer-reviewed clinical trials, particularly from bioactives derived from fish, mollusks, and crustaceans, compared to more extensively studied algal compounds. Preclinical data or in vitro evidence exist without sufficient in vivo or clinical validation, making it difficult to translate findings into safe commercial cosmetic formulations [57,78,113,519,520,521,522]. Additionally, variability in extraction methods, seasonal and species-based biochemical diversity, and inconsistent raw material sources can result in poor reproducibility and difficulties in standardization. Scalability and cost-effectiveness also remain concerns, especially for high-purity compounds like marine collagen peptides, fucoidans, or HA analogues derived from non-algal sources [57,78,519,520].

Another limitation is regulatory uncertainty. While databases like “Coslng” provide some listings, not all marine-derived ingredients have well-defined regulatory status, safety profiles, or toxicological data, especially those extracted from unconventional marine by-products. Furthermore, ethical and environmental concerns regarding marine biodiversity, bycatch utilization, and sustainable harvesting practices also require more transparent protocols and ecological assessments [28,78,79]. Finally, although cosmetic formulations using marine-derived bioactives demonstrate multifunctional properties ranging from moisturization to UV protection, a lack of consumer awareness and education might limit their market acceptance, particularly when compared to plant-based or synthetic counterparts [28,78,79].

The valorization of marine by-products for cosmetic and cosmeceutical utilization is a burgeoning field with tremendous potential. Future research should aim at deeper exploration, technological optimization, and regulatory consolidation to ideally support the industrial application of these marine-derived bioactives [78]. Future extraction and purification strategies must prioritize green chemistry principles, following the integration of green and sustainable technologies. This includes scaling-up of non-conventional extraction techniques such as EAE, MAE, UAE, SFE, and SWE, which may offer higher efficiency, reduced solvent use, and better preservation of thermolabile compounds. Hybrid (physical and enzymatic) processes could further enhance yield and bioactivity [57,113,519,521,522]. Recently, results proved that deep eutectic systems could also be a game changer for marine bioactives recovery [520]. Moreover, the variability of marine bioactives necessitates advanced analytical tools for characterization, like liquid chromatography (LC)–mass spectrometry (MS)/MS, nuclear magnetic resonance (NMR), and Fourier transform infrared spectroscopy (FTIR). Establishing standardized protocols for bioactive quantification, purity, and quality control will be critical in achieving batch-to-batch consistency and regulatory approval [57,519,520].

Encapsulation technologies are expected to play a transformative role in enhancing the cosmetic efficacy of marine-derived ingredients. Bioactives, including ω-3 PUFAs, carotenoids like astaxanthin, peptides, collagen hydrolysates, and chitosan derivatives, are often sensitive to degradation from UV radiation, oxidation, or extreme pH conditions. Advanced delivery systems, such as liposomes, nanoemulsions, micelles, solid-lipid nanoparticles, and polymeric nanoparticles, can protect these thermolabile compounds, increase their solubility, and enable controlled and targeted release on the skin. These delivery systems also improve bioavailability, minimize skin irritation, and allow the combination of multiple bioactives within a single formulation, thereby supporting more multifaceted and personalized cosmetic solutions [523,524,525].

Although numerous in vitro studies have demonstrated beneficial effects (e.g., anti-inflammatory, antioxidant, photoprotective), more mechanistic research is needed to unravel the biochemical pathways involved. This will allow the identification of molecular targets and structure–function relationships of marine bioactives, particularly peptides, PUFAs, and melanin derivatives [28,79,251]. Furthermore, longitudinal, randomized, and controlled trials are required to evaluate the bioavailability, efficacy, and safety of marine-derived ingredients when applied topically or systemically. Particular attention should be given to fish HA, collagen peptides, molluscan pigments, and chitosan derivatives from crustaceans. Formulations containing marine-derived bioactives must be tested for dermal penetration, pharmacokinetics, and adverse reactions [386,519,520]. Regulatory harmonization and consumer safety must also be addressed via the development of marine-specific ISO standards, ecolabels, and transparent databases [2,386,519,520].

The concept of a marine biorefinery, where different biomolecules (e.g., proteins, lipids, minerals, pigments) are fractionated from a single raw material, should be fully realized. By adopting circular economy models and zero-waste strategies, the cosmetic industry can not only benefit economically but also reduce the environmental burden of fishery waste [2,11,79,223,251,386,526]. Marine biotechnology, including microbial fermentation and synthetic biology, offers a sustainable pathway to replicate marine-derived bioactives without harvesting vulnerable species. For example, microbial expression of crustacean-derived chitosan or recombinant production of marine peptides could reduce ecological pressures while maintaining product consistency [527,528]. The modern cosmeceutical consumer is highly informed, sustainability-oriented, and demanding of both safety and efficacy. Future product development should incorporate marine bioactives into multifunctional, personalized formulations (e.g., UV-protective moisturizers with chitosan nanocarriers, anti-aging creams containing fish collagen peptides and astaxanthin) [2,386,519,520].

In summary, the next decade of marine cosmetic research will most likely focus on technological advances, encapsulation delivery systems, regulatory clarity, consumer education, and ecological responsibility. Collaborative efforts across academia, industry, and government sectors will be vital to unlock the full spectrum of benefits from marine-derived ingredients for human skin health and beauty.

## 6. Conclusions

Marine ecosystems represent a largely untapped reservoir of bioactive compounds with significant potential in cosmetic science. Marine-derived compounds from fish, crustaceans, and mollusks provide a wide array of biological benefits applicable to cosmetic applications, like skin, hair, nail, and oral care. These natural, biocompatible, and sustainable bioactives exhibit notable anti-inflammatory, antioxidant, anti-aging, skin-whitening, antimicrobial, UV-protective, and moisturizing effects, making them attractive candidates for incorporation into creams, lotions, ointments, serums, masks, and sunscreens as alternative solutions to synthetic ingredients.

The extreme conditions of marine environments, such as high salinity, pressure, and UV exposure, favor the biosynthesis of structurally unique and biologically potent compounds. Therefore, the valorization of marine by-products like fish skin, scales, and bones, crustacean shells, and mollusk extracts aligns with the principles of circular economy and sustainable cosmetics. However, while considerable progress has been made in identifying and characterizing these compounds, further research is required to optimize their extraction, validate their efficacy through clinical studies, and ensure regulatory compliance. As consumer interest shifts toward natural, eco-friendly, and functional skincare, marine bioactives offer a promising and innovative toolkit for developing next-generation cosmetic products.

## Figures and Tables

**Table 1:** Challenges and opportunities of fishery bycatch and by-products (Adapted from multiple sources, including [37,48,49]).

Challenges	Benefits
Negative environmental impact due to discards and waste accumulation	Promote fishery sustainability and enable environmentally friendly waste management
Loss of high-value bioactives through discarding heads, skins, viscera, bones, etc.	Valorization of compounds like enzymes, lipids, and peptides for high-end uses
Lack of standardized extraction protocols	Advances in green and selective extraction methods (e.g., enzymatic hydrolysis, membrane filtration)
High operating and processing costs for waste handling	Resource-efficient use through biorefinery models and circular economy integration
Short shelf-life and high perishability due to lipids and enzymes	Stabilization techniques improve raw material storage for long-term industrial use
Regulatory restrictions for classifying and reusing by-products	Growing acceptance and guidance under the EU Circular Economy Action Plan (CEAP) and the Food and Agriculture Organization (FAO) sustainability goals
Consumer perception issues regarding waste-derived ingredients	Increasing demand for natural, marine-origin, clean-label bioactives in cosmetics and supplements

**Table 2:** Advantages and disadvantages of the most frequently used extraction methods in fishery bycatch and by-products.

Extraction Methods	Advantages	Disadvantages	References
Enzyme-assisted extraction (EAE)	Nontoxic, environmentally friendly High yield of bioactives (e.g., polysaccharides, peptides) Converts insoluble materials into water-soluble Mild conditions, suitable for thermolabile compounds Inexpensive (uses food-grade enzymes) Applicable to marine algae, mollusks, and fish tissue	Extended processing time (several hours to days) Optimization required for pH, enzyme type, and ratio Limited penetration in dense matrices Enzymatic residues may need removal	[54,72]
Microwave- assisted extraction (MAE)	High extraction efficiency Short processing time and low solvent use Effective for polyphenols, pigments, and oils Good for crustacean shells	Risk of degradation of thermo-unstable compounds High energy consumption Requires solvents with strong dielectric properties Uneven heating at larger scales	[73,74]
Subcritical water extraction (SWE)	Green method (uses only water as a solvent) Efficient for both polar and non-polar compounds No organic solvent residue Suitable for proteins, polyphenols, and sugars	High-pressure apparatus is costly Requires corrosion-resistant equipment Not ideal for thermosensitive compounds Limited scalability	[75]
Supercritical fluid extraction (SFE)	High selectivity for targeted bioactives Solvent-free final extract (e.g., CO2 evaporates completely) Low temperatures, ideal for thermolabile compounds Suitable for oils, carotenoids, and sterols Clean and efficient for fish and crustacean lipids	High setup and maintenance costs Needs high pressure (above 74 atm) For polar chemical compounds, toxic modifiers (e.g., methanol) may be needed Complex instrumentation Longer extraction time	[76]
Ultrasound-assisted extraction (UAE)	Short extraction time, minimal solvent and energy use Works well with marine algae and fish skin Elevated yield of proteins, pigments, and polyphenols High efficiency in cell disruption (cavitation effect) Compatible with other methods (e.g., UAE-EAE)	Limited to solvents with low viscosity and vapor pressure Over-sonication may degrade sensitive compounds Risk of equipment wear due to cavitation Less effective on hard biomass (e.g., shells)	[70,77]

**Table 3:** Valuable bioactive compounds derived from fish processing by-products.

Fish By-Product	Percentage (% *w*/*w*)	Valuable Compounds	References
Skin	3.5	Collagen, gelatin, antimicrobial compounds (e.g., cathelicidins, defensins), and ω-3 fatty acids	[37,174,192]
Bones	10–15	Calcium phosphate (hydroxyapatite), collagen, gelatin, ω-3 fatty acids, lipids, and P	[37,42,43,243]
Scales	2	Collagen, chitin, chitosan, minerals (e.g., Zn, Ca), and glycoproteins	[37,244]
Head	10–15	Protein hydrolysates, enzymes (e.g., pepsin, trypsin), bioactive lipids, gelatin, and bioactive peptides	[175,198,245,246,247]
Viscera	12–18	Fish oil (e.g., EPA, DHA), digestive enzymes (trypsin, lipase), probiotics, bile acids, and vitamin D	[37,175,248]
Swim bladder	39.2	Type I and II collagen, gelatin, GAGs, and elastin	[240,249,250]
Fins	~1–2% (estimated *)	CS, HA, and collagen	[239,240]
Cartilage	- *	CS, GS, and protein–polysaccharide complexes	[240,241]
Liver	~5–15% (estimated *)	Vitamins A and D_3_, ω-3 PUFAs, and antioxidant enzymes	[186,192,240]

* Percentages are estimated based on species and processing method. Exact values for fins, cartilage, and liver are not widely standardized in the literature. (Important data of this table were obtained from [44].

**Table 4:** Examples of commercial cosmetic products utilizing marine by-product ingredients. (Commercial examples listed mainly in Scopus-indexed databases, recommended products, and https://cosmetics.specialchem.com (accessed on 24 June 2025).)

Marine Source	Patent Name or Cosmetic Formulation	Marine Bioactives (Formulation Content)	Cosmetic-Associated Benefits and Commercial Use	References
Fish	Marine Hydrolyzed Collagen Imw^TM^ (Ashland Global Holdings, Wilmington, DE, USA)	Marine collagen oligopeptides upcycled from fish skin	Biofunctional cosmetic ingredient for scalp and hair purposes Collagen boosting and glow-enhancing results Use in skin-care, hair-care, makeup, and bath products	[251]
	Affinisphere^TM^ (BASF, Ludwigshafen, Germany)	Marine Atelocollagen microspheres from shark fins	Oily skin improvement Perfect for impure and oily skin during summer and humid climates Mattifying cream and serum products	[251]
	Marine Filling Spheres^TM^ (BASF, Ludwigshafen, Germany)	Dehydrated microspheres of marine collagen and GAGs from shark fins	Moisturization and anti-wrinkle properties Skin soothing and surface-smoothing results Use in anti-aging, facial skin health, and lip-care products	[251]
	COLLASURGE^TM^ (Croda Beauty, Yorkshire, UK)	Marine collagen amino acids from fish species	Moisturizing, hydrating, nourishing, repairing, soothing, cooling, and softening properties for creams, gels, skin- and hair-care products	[251]
	ICHTYOCOLLAGEN^TM^ (Sederma by Croda Beauty, Snaith, UK)	pH-soluble marine collagen from fish skin extract	Moisturizing, hydrating, nourishing, repairing, and film-forming properties Used in creams and gels	[251]
	Finn Canada (Canada)	FINN’s salmon skin collagen (Salmonollagen)	Improves skin condition, treating wrinkles, spots, dryness, dullness, and acne Used in cleansing foams, scrub cleansing foams, gels, creams, extracts, toning, and milk-lotions	[28]
	PURE MARINE COLLAGEN (Kenney and Ross Limited, Port Saxon, Nova Scotia, Canada)	Hydrolyzed collagen from the skin of deep-sea, wild fish	Promotes healthy skin, nails, and hair outcomes Improves skin hydration, elasticity, and firmness Used as a protein additive in cosmetics, nutraceuticals, or food products	[28]
	Collagen HM^TM^ Sol, Elastin TM^TM^, Glycosann^®^ Sol, and Protein M+^TM^ (220 Rue du Petit Port, France)	Hydrolyzed marine collagen, elastin, chondroitin sulphate, and cartilage extract from fish by-products	Moisturizing, cell firmness, regeneration, and elasticity benefits for anti-aging and anti-wrinkle purposes	[252]
	ThalaCol^TM^ (Thai Union Ingredients (Global), Bangkok, Thailand)	Contains 100% pure hydrolyzed marine collagen peptides from wild tuna skin	Provides more radiant and youthful skin, offering effective incorporation into topical skincare cosmetics like moisturizer sticks	[253]
	NUTRICOLL NATURAL MARINE COLLAGEN POWDER (Chitinor, Seagarden), Norway)	Natural fish collagen peptide derived from wild cod	Anti-aging, anti-wrinkle, moisturizing, and hair protection bioactive, free from artificial additives and preservatives Used in face masks, creams, serums, lotions, hair balms, shampoos, and conditioners	[254]
	Cropure^TM^ Orange Roughy (Croda Beauty, UK)	Orange roughy oil (*Hoplostethus atlanticus*)	Good-spreading, non-greasy, emollient, softening, and moisturizing agent in creams, lotions, sun-care, color, and bath cosmetics	[255]
	Zellulin^®^ ZelluGEN^TM^ 10% Solution (Avant, Old Street, London)	Hydrolyzed minnow muscle cell lysate biopeptides	Regenerating, revitalizing, skin-smoothing agent used in leave-on, body care, and cosmetic products Anti-aging agent that promotes elastic skin, collagen production, and skin hydration benefits	[256]
Crustaceans	HYDAMER^TM^ HCMF^TM^ (Chitinor^TM^, Tromsoe, Norway)	Chitosan from crustacean chitin	Film-forming and moisturizing agent in cosmetics Crack-free film that offers natural, flexible hold and elasticity without stickiness or added heaviness Used in hair setting and gels, styling mousses and creams, curl retention, hair-tip fluids, anti-dandruff preparations, hair tonics, and conditioners	[257]
	EARTHEN InstantPeel^TM^ Exfoliant (EARTHEN SkinCare^TM^, Pleasantville, New York)	Shrimp extract, fish oil, and hydrolyzed collagen from fish	Gentle exfoliation and moisturization properties Exfoliant aimed to improve skin texture (smooth, soft, and luminous-looking)	[258]
	System4^®^ R (Finland)	Chitosan from marine crustacean exoskeleton	Hair smoothing, moisturizing, and perfume-free products Leave-in chitosan spray Lightweight consistency for both normal and oily hair	[259]
	DHC Astaxanthin Power Cream (DHC, USA)	Astaxanthin is found in large amounts in the shells of crustaceans and salmon	Lightweight, fast-absorbing, facial moisturizer that tones, hydrates, and brightens the skin for achieving a radiant-looking skin complexity	[260]
	Marin Soothing Hydration Cream (Marin Skincare, ME, USA)	Marine lobster glycoproteins	Skin-soothing and anti-irritating agents that increase natural hyaluronic acid production and overall skin cell viability Soothing hydration cream (lobster lotion) and lip treatment products	[261]
Mollusks	CRODAROM^®^ BLACK PEARL (Croda Beauty, Yorkshire, UK)	Aragonite and conchiolin from Tahitian black pearls from the black-lip oyster (*Pinctada margaritifera*)	Revitalizing, moisturizing, and nourishing properties Protecting skin against environmental damage Suitable for decorative cosmetics, radiance creams, or gloss shampoos	[251]
	Creanatural^TM^ SEPIA MELANIN (The Innovation Company^®^, France)	Creanatural Sepia Melanin derived from squid Ink	Photoprotective and antioxidant properties Effective UV filter for skin, sun, and hair-care cosmetics	[78]
	Akoshine^®^ (Akott Evolution, Milan, Italy)	Oyster shell pearl powder	Moisturizing, sun-protective, anti-aging, and smoothing product that provides a soft and silky touch for creams, gels, lotions, massage formulations, body talc, tonics, powders, nail creams, and foundations	[262]
	Oligoceane^TM^ PH (Croda Beauty, UK)	Oyster shell extract and sea salt extract	Nourishing, moisturizing, conditioning, and skin-repairing agent in after-sun, body-, face-, neck-, skin-, and foot/leg-care products	[263]
	Huzhou Pearl Powder (Huzhou Shengtao Biotech, Huzhou Zhejiang, China)	Amino acids and minerals from the jib clam of a natural naidinae animal or the wrinkle clam of a bivalve mollusk	Anti-inflammatory, anti-wrinkle, whitening, and skin-healing/smoothing properties Eliminates black spots and can slow down senile wrinkles in skin	[264]

**Table 5:** Skin-related cosmetic patents containing MAAs in their formulation.

Patent Name	Skincare Product	MAA Components/Formulation Content	Skin-Associated Benefits	References
WO2024027929A1	Sunscreen	Mycosporine-like amino acid compounds (the sunscreen contains organic heterocyclic compounds)	Improved water resistance Increased SPF performance, without being sticky Anti-UV and antioxidant action	[400]
WO2024028510A1	Sunscreen	Mycosporine-like amino acid compounds (formulation that contains heterocyclic organic compounds)	Improved SPF performance UVA (and UVB) photoprotection Anti-aging, whitening, or skin-darkening preventative activity Enhanced skin radiance and firmness	[401]
CN105342903A	Biological sunscreen	The formulation contains 70–85 parts of MAAs, 0.5–5 parts UV light absorber, and 10–15 parts of a biological pigment	Effectively enhances UV protection and skin brightness Free of chemical irritation (biological) Anti-allergic and UV-proof effects	[402]
CN103720625A	Sunscreen	MAAs from seaweed polyphenols (Sargassum polysaccharides) The formulation contains 0.1–10 parts of Sargassum polysaccharide, parts of MAAs, and a Sargassum polyphenol mixture	UV absorption capability Free radical scavenging activity and reducing power benefits Moisturizing effect (skin hydration enhancement), good spreadability, and a relatively refreshing, non-sticky sense after use	[403]
CN106937918A	Cosmetic emulsion	MAAs, phycobiliproteins, polysaccharides, and seaweed polyphenols The formulation contains 0.01–3 parts of the class mycetocyte element amino acid, 0.1–4.5 parts of phycobniliprotein, 0.01–2.5 parts of laver amylose, 0.1–2.4 parts of Tea polyphenols	Absorbs UVA, UVB, and electromagnetic radiation Neutralizes free radicals and suppresses lipid peroxidation, offering antioxidant benefits Offers high flexibility, reducing radiation-induced skin irritation Improves the self-protection ability of the skin	[404]
WO2023004522A1	Water-in-oil-water (W/O/W) nanoemulsion	Curcumin and Pyropia columbina seaweed extract rich in MAAs	Photo-stability and anti-UV effects Mitigates and repairs cell damage Beneficial for dermatological use	[405]
WO2024027926A1	Sunscreen	MAAs, along with a photo-unstable UV filter, and as a stabilizer	Improves the photo-stability of a photo-unstable UV filter Enhances UV-absorption	[406]
KR20170090690A	Wound-healing composition	MAA powder mixture with purified water	Skin regeneration, damage restoration, and wound-healing	[407]
CN105310897A	Anti-freeze hand cream	The formulation contains 10–20 parts of MAAs, 40–60 parts of an emulsifier, 1–10 parts of an antioxidant, and 10–15 parts of allantoin	No reported chemical irritation (nontoxic, biological) Offers UV protection Successfully prevents frost cracks	[408]
WO2024027930A1	Antimicrobial boosting agent	Antimicrobial agent together with MAAs	Improvement in microbial stability during storage	[409]

**Table 6:** Examples of amino acid chain (small peptides) skin-related functions and commercially utilized products.

Amino Acid Sequence (Small Peptide Chain)	Skin-Related Mechanism of Action	References
Valine–glycine–valine–alanine–proline–glycine (VGVAPG, Palmitoyl oligopeptide)	Triggers collagen synthesis and reduces elastin synthesis Palmitic acid enhances chemo-attraction for skin fibroblasts Palmitic acid enables oligopeptide epidermis penetration Enhances skin elasticity and reduces wrinkle appearance Proven antioxidant value (ROS scavenging)	[382,433,434,435]
Lysine–threonine–threonine–lysine–serine (KTTKS and with palmitic acid: Palmitoyl Pentapeptide-4, Matrixyl^®^)	Acts as a subfragment of procollagen type I Benefits the production of collagen types I, II, III, and IV, elastin, fibronectin, and GAGs, reducing skin roughness and wrinkles Inhibits collagenase activity, promoting wound-healing Palmitic acid enhances its stability and enables skin penetration	[382,435,436,437]
Tyrosine–tyrosine–arginine–alanine–aspartame–aspartame–alanine (YYRADDA)	Inhibits procollagen C-proteinase by cleaving the C-pro-peptide from procollagen-1 Potential reduction of collagen breakdown	[382,435,438]
Lysine–phenylalanine–lysine + elaidic acid (Lipospondine) (lysine–valine–lysine functions similarly + palmitic or bistrifluoroacetic acid)	Activates the latent transforming growth factor β (TGF-β) Inhibit collagenase via its lipophilic moiety (elaidic acid) Increase collagen and reduce collagenase levels Lysine–valine–lysine is currently marketed under the brand name of Palmitoyl tripeptide-3/5	[382,435,438]
Phenylalanine–valine–alanine–proline–phenylalanine–proline (Peptamide^®^-6)	Exerts wound-healing properties and improves collagen synthesis Influences skin growth factors powerfully Upregulates gene expression of the ECM and other cell stress genes, increasing skin firmness	[382,435,438,439]
Glycyl-*L*-histidyl-*L*-lysine	Increases collagen formation by triggering fibroblasts Stimulates type I collagen and certain GAGs like dermatan sulfate and heparan sulfate Mixed with palmitic acid, it decreases the depth and length of wrinkles, makes the skin smoother, and improves texture Modulates anti-aging molecular pathways in sensitive skin types Increases tissue inhibitors of MMP1 and MMP2 levels	[382,435,438]
Heptapeptide (aspartic acid–glutamic acid–glutamic acid–threonine–glycine–glutamic acid–phenylalanine–DEETGEF-OH (Perfection Peptide P7^TM^)	Protects cell DNA, stimulating antioxidant enzymes Offers protection to skin cells against UV damage by triggering nuclear factor erythroid 2-related factor 2 (Nrf2)-dependent antioxidant enzymes Anti-aging and photoprotective potential	[435,440]
Asparagine–tyrosine–arginine–arginine–glutamic acid (NYRRE) and arginine–histidine–alanine–lysine–phenylalanine (RHAKF)	RHAKF exhibited stronger superoxide anion radical scavenging activity and lipid peroxidation inhibition than NYRRE RHAKF also displayed better copper chelating activity, tyrosinase inhibition, and thus, skincare potential	[441,442]
Oligopeptide-68 (β-white, arginine–aspartic acid–glycine–glutamine–isoleucine–leucine–serine–threonine–tryptophan–tyrosine)	Utilized as a whitening agent for melasma-affected skin Antioxidant, anti-aging, moisturizing, anti-inflammatory, and collagen-stimulating properties Potential skin pigmentation contributors	[435,443]
Acetyl tripeptide-30 citrulline	Utilized in wrinkle-smoothing formulations Exerts anti-inflammatory and antioxidant benefits in skin cells Upregulates collagen IV and downregulates MMP9, enhancing the expression of skin barrier proteins (anti-aging benefits) Increases the expression of aquaporin 3, improving skin hydration	[444]
Pentapeptide 3 (Vialox^®^)	Softens wrinkles and decreases skin roughness Competitive antagonist of acetylcholine receptors	[435,438]
Pentapeptide-18 (Leuphasyl^®^)	Blocks calcium channels in the neuron, inhibiting acetylcholine release (functions like enkephalins) Reduces wrinkle depth and volume	[435,438]
Acetyl-glutamyl-glutamyl-methoxil-glutaminyl-arginyl-arginylamide (Argireline^®^)	Inhibits neurotransmitters’ release, affecting the generation and stabilization of the protein complex necessary for docking vesicles of acetylcholine release (nontoxicity) Creams with this peptide reached a 27% reduction in periorbital wrinkle depth in female subjects	[435,438,445]

**Table 7:** Examples of marine peptides: cosmetic-related functions and applications.

Marine Type	Marine Species	Bioactive Peptides	Biological Activity	Cosmetic-Related Functions	References
Fish	*Tilapia*	Skin-collagen peptides	Oral mucosal ulcers	75% marine collagen peptides promoted the proliferation and migration of L929 cells Enhanced ulcer healing Suppressed inflammation Upregulated expression of the vascular endothelial (VEGF) and fibroblast growth factors (FGF)	[454]
Fish	Tuna (*Thunnus obesus*)	Collagen peptide from skin (TSCP) and bone (TBCP)	UV_B_ protection, photo-aging, and radical scavenging/antioxidant effects	Reduced skin photo-aging via MAPK and TGF-β signaling pathways Inhibited phosphorylated (p)-p38 activation, enhanced TGF-β1, and decreased p-ERK, p-Janus kinase inhibitor (JNK), and MMP-1 ROS scavenging antioxidant action Improved cell viability, suppressed mRNA and protein expression Inhibited skin water loss (TEWL)	[455]
Fish	Black pomfret (*Parastromateus niger*)	Peptides	Anti-aging potential	Improved skin aging due to the free-radical-scavenging and anti-aging activity of the peptides	[448]
Fish	Hydrolyzed fish cartilage	Collagen peptides	Anti-aging potential	Reduced wrinkles and increased dermis echogenicity Enhanced collagen morphology and decreased elastosis Improved morphological skin characteristics and thickness	[456]
Fish	Giant croaker (*Nibea japonica*)	Swim bladder collagen peptides: acid- and pepsin-solubilized collagen (ASC and PSC)	Wound-healing and Antioxidant potential	ASC and PSC displayed antioxidant and radical-scavenging properties Collagen sponge peptides accelerated wound-healing (scratch closure rate) Decreased IL-1β, IL-6, and tumor necrosis factor α (TNF-α) levels (anti-inflammatory benefits)	[457]
Fish	Sea bass (*Late calcarifer*)	Hydrolyzed collagen from the defatted Asian sea bass (Asbs-HC)	Antioxidant, skin nourishment, and wound-healing potential	Pro-Hyp and Pro-Hyp-Gly were the peptides present in the sea bass Asbs-HC (1000 μg/mL) showed the best cell proliferation and migration Accelerated the wound-healing process by increasing fibroblast mobility at the site of the injury	[458]
Fish	Crimson snapper	Crimson snapper scale peptides (CSSPs)	Antioxidant and anti-aging potential	Enhanced the antioxidant enzyme activity and upregulated the expression of antioxidant genes Prolonged the mean lifespan of skin	[452]
Fish	Salmon and codfish skin	Collagen peptides	Anti-aging and skin-related health potential	Revealed good moisture absorption Prevented skin dehydration Presented nontoxic and non-irritating outcomes	[28]
Fish	Chum salmon skin	Marine collagen peptides (MCPs)	Wound-healing and anti-angiogenesis potential	Accelerated tissue regeneration at the wound site (wound-healing) Improved angiogenesis Formed thicker and more organized collagen deposition	[459]
Fish	Salmon skin	Gelatin and hydrolysates	Anti-aging, anti-UV, and anti-photo-aging potential	Improved UV-irradiation-induced pathological alterations in skin texture and morphology Decreased hydroxyproline content in photo-aging skin Increased SOD, CAT, glutathione (GSH), and its peroxidase (GSH-PX) (antioxidant potential) Participated in collagen synthesis	[428]
Fish	Atlantic salmon egg extract (*Salmo salar*)	Serine endoproteases, oleic and linoleic acid	Anti-wrinkle, anti-aging, and skin-health-promoting	Improved wrinkles, pigmentation, redness, brightness, and hydration Neither skin irritation nor sensitization were observed	[460]
Crustacean	Shrimp shell	Chitosan oligosaccharide	Anti-aging and antioxidant potential	Increased SOD, CAT, GSH, and (GSH-PX) (antioxidant potential) Decreased malondialdehyde levels (MDA) and increased serum immunoglobulin G (IgG) Inhibited D-galactose upregulation of alanine aminotransferase (ALT), aspartate transaminase (AST), alkaline phosphatase (ALP), uric acid (UA), and creatinine levels	[461]
Crustacean	Crab (*Portunus sanguinolentus*)	Crab chitin nanofibrils	Anti-UV_B_ and photoprotective potential	Protected against UVB irradiation Anti-inflammatory, antioxidant, and photoprotective benefits	[462,463]
Mollusk	Oyster (*Crassostrea hongkongensis*)	Protein enzymatic hydrolysates (WNLNP and RKNEVLGK)	Anti-photo-aging skin potential	Enhanced dose-dependently the anti-photo-aging effect on UVB-irradiated HaCaT cell lines WNLNP inhibited ROS and reduced MMP-1 expression Elevated pro-collagen I synthesis Suppressed p38, JNK, ERK, and p65 phosphorylation in the MAPK/NF-κΒ signaling pathway Reversed B-cell lymphoma 2 protein (Bcl-2) reduction in UVB-irradiated HaCaT cells	[447]
Mollusk	Squid (*Symplectoteuthis oualaniensis*)	Acid- and pepsin-solubilized collagen peptides (ASC and PSC)	Stem-cell regenerative and skin-cell protective effects	Enhanced human foreskin fibroblast 1 (HFF-1) proliferation and migration Restored the proliferation of HFF-1 cells damaged by H_2_O_2_	[464]

**Table 8:** Examples of astaxanthin sources, delivery systems, and skin-related functions.

Study Hypothesis	Administration Pattern	Cosmetic and Cosmeceutical Benefits	References
Randomized and double-blind study of 65 healthy women exposed to UV_B_ radiation	Marine-derived AST capsules at 6 or 12 mg/day for 16 weeks or a placebo	AST suppressed UV_B_-induced inflammatory cytokine secretion in keratinocytes and MMP-1 secretion in fibroblasts Attenuation of wrinkle formation and skin elasticity enhancement AST supplementation may inhibit age-linked skin deterioration and maintain skin conditions	[493]
Randomized and double-blind study of 23 healthy Japanese participants exposed to UV_B_ radiation	Algal-derived AST capsules at 4 mg for 9 weeks or a placebo	Increased MED compared to the placebo Reduced loss of skin moisture in the irradiated area compared to the placebo Improved rough skin and texture in non-irradiated areas Protective effects against skin deterioration and skin health maintenance	[485]
Morphological analysis in residual skin surface components (RSSCs) for monitoring oxidative stress and skin aging in 31 middle-aged volunteers	AST capsules at 4 mg for 4 weeks (results before and after the administration)	Consistently decreased plasma MDA during AST consumption (by 11.2% on day 15 and 21.7% on day 29) Notably decreased levels of corneocyte desquamation and microbial presence A significant increase in lipid droplet size was also manifested among obese subjects Continuous AST consumption offers strong antioxidant and skin rejuvenation benefits	[494]

**Table 9:** Summary table of marine bioactives, their sources, and skin-related benefits.

Family of Compounds	Bioactive Compounds	Skin-Health-Related Function	Marine Organism	References
Polysaccharides	Chitin, chitosan, and derivatives, hyaluronic acid, GAGs, and fucoidans	Anti-pigmentation, antibacterial, moisturizing, antioxidant, anti-aging, MMP-inhibitory, anti-cancer, anti-inflammatory, antifungal, UV-protection, skin-cleansing, nail-care, hair-care, oral-cavity-care, gel-forming, skin-rejuvenant, and wound-healing properties	Exoskeleton of crustaceans, marine gastropods, fish, fish mucus, and mollusk tissues	[11,28,37,78,117,265,266]
Fatty acids	SFAs, MUFAs, and mainly PUFAs (e.g., LA, DHA, EPA, ALA, arachidonic acid)	Collagen stimulation, emollient, anti-inflammatory, anti-inflammatory, antioxidant, hydrating, anti-photo-aging, anti-melanogenesis, dermatitis-protective, anti-cancer, wound-healing, anti-hyperpigmentation, anti-erythema, anti-psoriasis, and acne vulgaris-protective properties	Fish head, oil, frame, trimming, viscera, skin of salmon and tuna, several fatty fish, and crustaceans like crab, mussels, and oysters	[11,28,37,78]
Lipid bioactives	Polar lipids like phospholipids, glycolipids, sphingolipids, and sulfolipids (e.g., marinosomes, lecithin) and lipid vitamins (e.g., A, B, D, E, or K)	Anti-inflammatory, emollient, hydrating, wound-healing, barrier-repairing, delivery-enhancing, photoprotective, restructuring of cell membranes, antioxidant, anti-aging, emulsifying, collagen-stimulating, and anti-skin-related diseases properties	Marine organisms, including microalgae, krill, sea cucumbers, mollusks, crustaceans, and fish by-products like oil, roe, liver, viscera, or skin	[11,78,147,320,364,365,366,373,374,375]
Amino acids	All 20 standard residues and mostly MAAs	Moisturizing, smoothing, youthful-looking skin-promoting, anti-aging, anti-allergic, photoprotective, wound-healing, collagen-stimulating, skin elasticity and firmness-enhancing, emulsifying, UV-protective, antimicrobial, antioxidant, anti-inflammatory, anti-wrinkle, exfoliation efficacy, anti-melanogenic, skin-soothing, and anti-irritating properties	Marine and freshwater organisms like cyanobacteria, fungi, and algae and higher-order creatures like cnidaria, fish, arthropods, tunicates, sponges, echinoderms, and mollusks	[11,78,383,389,390,391,392,393,394,498]
Proteins	Collagen, elastin, and gelatin	Antioxidant, anti-aging, anti-wrinkle, moisturizing, structural support, skin-whitening, UV-protection, collagen-stimulating, hydrating, water-binding, anti-melanogenic, skin-soothing, and anti-inflammatory properties	Fish bones, skin, scale, and swim bladder, mollusks, and crustaceans	[11,18,28,37,78,192,410,415,417,418,419,459]
Peptides	Small amino acid chains, collagen and gelatin hydrolysates	Anti-aging, antioxidant, anti-inflammatory, oxygen synthesis stimulation, elasticity restoration, collagen-stimulating, anti-wrinkle, skin firmness-enhancing, texture-improving, UV-protective, photoprotective, radical-scavenging, tyrosinase-inhibitory, skin-whitening, moisturizing, MMP-inhibitory, immunostimulant, antibacterial, and anti-cancer properties	Fish muscle, skin, heads, and trimming waste, hydrolyzed fish cartilage, shrimp shells, crabs, oysters, and squids	[11,37,78,382,435,438]
Carotenoids and pigments	Astaxanthin, melanin (allomelanin, neuromelanin, eumelanin, pheomelanin, and pyomelanin), lutein, β-carotene, halocynthiaxanthin, and fucoxanthin	Antioxidant, anti-inflammatory, photo-protective, skin pigmentation-regulating, cosmetic preservative, antioxidant, anti-UV, radical-scavenging, moisturizing, anti-wrinkle, UV-ray-protecting, anti-tumor, anti-eczema, and wound-healing properties	Crustaceans, shrimp head, shell, and tail, freshwater fish or red fish, Japanese mackerel and amberjack (yellowtail), rainbow trout, cephalopod ink, and mollusks	[11,28,78,192,473,495,496,515,516]
Phenolic compounds	Polyphenols, PBDEs, and phlorotannins (e.g., diphlorethol, triphloroethol, trifuhalol, tetrafuhalol, phloroglucinol, eckol, and eckstolonol)	Antioxidant, anti-inflammatory, anti-cancer, UV-protective, radical-scavenging, hydrating, anti-aging, anti-tumor, anti-eczema, wound-healing, anti-melanogenesis, and anti-hyperpigmentation properties	Fish, crustaceans, bivalves, shrimps, clams, oysters, mussels, red and brown algae	[78,192,502,503,504,505,506]
Substrates and minerals	Hydroxyapatite, CaPs, minerals like Ca, Mg, and Zn, and powdered pearl shells or nacreous shell layers	UV-protective, skin and teeth remineralization, wound-healing carrier, skin rejuvenation, skin cell proliferation, anti-inflammatory effects, anti-acne, sebum-modulating, anti-age-related degenerative disorders, skin tone-enhancing, skin fibroblast-regulating, moisturizing, collagen-rebuilding, angiogenesis-promoting, wound-healing, whitening, deodorizing, and antibacterial cosmetic properties against hyperpigmentation, atopic dermatitis, deep-burn porcine skin, burn scars, and skin cancer incidence	Fish bones, teeth of vertebrates, shells from crustaceans, mollusks, and pearls from oysters and mussels	[28,37,39,78,79,192,509,510,511,512,513,514]
